# Alzheimer’s Disease Accelerates Cerebral Atrophy by Over a Decade Compared to Healthy Aging

**DOI:** 10.1007/s10439-026-04059-z

**Published:** 2026-03-24

**Authors:** Shima Jalalian, Johannes Weickenmeier

**Affiliations:** 1 Department of Mechanical Engineering, Stevens Institute of Technology, Hoboken, NJ 07030 USA; 2 Department of Engineering Science, University of Oxford, Oxford, OX3 7DQ UK

**Keywords:** Cerebral atrophy, Cognitively normal, Mild cognitive impairment, Alzheimer’s disease, Multiphysics finite elements, Aβ and tau coupling

## Abstract

Brain aging is accompanied by progressive morphological and neurobiological changes, which are significantly accelerated in neurodegenerative diseases, such as Alzheimer’s disease. Detecting and differentiating these changes early is crucial for diagnosis, treatment planning, and therapeutic development. In this work, we present a computational multiphysics framework that couples protein biomarker propagation with tissue-level atrophy to distinguish between cognitively normal aging, mild cognitive impairment, and Alzheimer’s disease. Our model integrates a network-based simulation of amyloid beta and tau protein spread with a finite element model of brain mechanics to simulate longitudinal brain shape changes over 40 years. Notably, we observe that amyloid beta accumulation precedes tau-driven degeneration by over a decade, aligning with empirical biomarker studies. We also introduce several mechanomarkers which are quantitative metrics of brain morphology such as displacement, cortical thickness, curvature, and sulcal depth. They serve as quantitative measures of disease-specific deformation patterns. Our simulations predict that Alzheimer’s disease accelerates cerebral atrophy by about 12 years relative to normal aging, with early divergence in medial temporal and occipital regions. Our findings identify cortical thickness and area stretch as early and sensitive markers to distinguish between healthy and abnormal aging. Spatially, the supramarginal gyrus and entorhinal cortex should be considered as regions of early vulnerability. These results underscore the potential of physics-informed computational models to improve early detection of neurodegeneration and guide the development of region- and stage-specific diagnostic tools.

## Introduction

Brain aging, both in cognitively normal and in the context of neurodegenerative conditions like Alzheimer’s disease, involves significant structural and neurobiological deterioration [1, 2]. Functionally, aging is accompanied by cognitive decline that varies significantly between subjects but is exacerbated by neurodegeneration [3]. There has been many efforts of trying to link, biological, structural, and functional changes during aging with the goal to develop either earlier diagnostic tools or support the development of neuroprotective interventions. Importantly, there is a noticeable temporal delay between the onset of structural changes and functional decline. Given the limited access to brain tissues for diagnostic purposes, however, it remains challenging to precisely detect brain shape changes during early stages of abnormal aging.

**Fig. 1:**
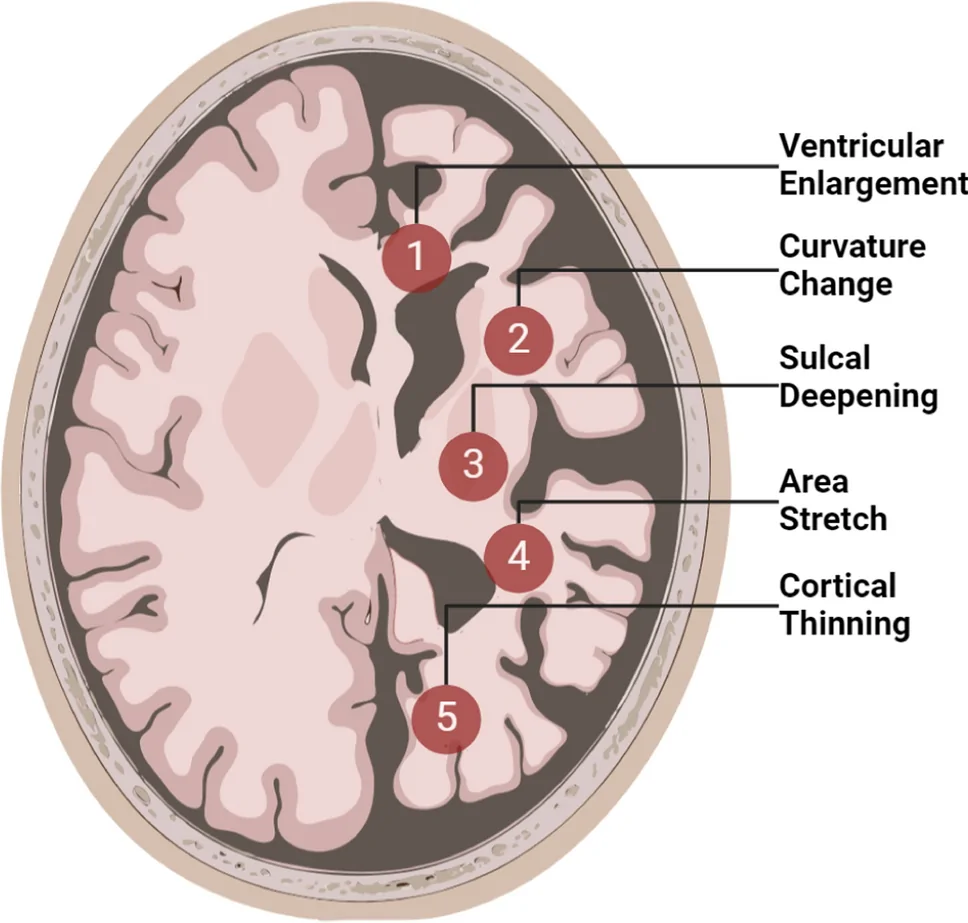
Comparison of healthy-looking brain hemisphere (left) and aging-driven cerebral atrophy (right) which shows the hallmark features of cerebral atrophy including ventricular enlargement, sulcal depth change, area stretch and curvature of functional brain surfaces, and cortical thinning

Figure1 compares a healthy brain hemisphere (left) and a hemisphere showing the characteristic manifestations of cerebral atrophy, including ventricular enlargement, alterations in cortical surface curvature, increased sulcal depth, area stretch, and progressive cortical thinning (right) [2]. These highly reproducible findings in the aging brain—and especially the rate at which they change with age—may serve as a marker to distinguish between healthy and abnormal aging. Pathologically, the progression from normal aging to mild cognitive impairment and Alzheimer’s disease is primarily driven by the joint accumulation of two neurotoxic protein concentrations: extracellular amyloid beta plaques, or Aβ, and intracellular neurofibrillary tangles, or tau [4, 5]. Only imaging-, blood-, or spinal fluid-derived measures allow for quantifying the state of the brain [6, 7]. Unfortunately, most of these measures are invasive and, for that reason, challenging to follow longitudinally [8, 9]. This makes their use in clinical practice unreasonable and warrants search for alternative methods.

Over the past several years, many computational models have emerged that, together with physics-based constitutive models, provided insightful simulations into the spatiotemporal progression of aging and neurodegenerative diseases [10–15]. As such, we propose introducing a refined multiphysics model with the distinct goal to distinguish healthy from abnormal aging in order to eventually influence clinical decision-making and patient care planning [16].

The objective of the present work is to establish an integrated multiphysics framework that bridges the gap between protein propagation and tissue-level mechanical changes associated with cognitively normal aging, mild cognitive impairment, and Alzheimer’s disease. Additionally, we propose a constitutive model that couples Aβ and tau biomarker concentrations to inform location-specific and age-dependent atrophy rates. We build on our previous model that coupled a single biomarker concentration to cerebral atrophy [13, 17]. Here, we extend this existing model to allow for incorporating the intricate interplay between Aβ and tau in predicting the progression of neurodegeneration. To that end, our model distinguishes between healthy aging associated with cognitively normal subjects and accelerated aging, i.e., neurodegeneration associated with mild cognitive impairment and Alzheimer’s disease. Our simulations predict not only the spreading of toxic proteins across the brain but the corresponding deformation resulting from white and gray matter tissue degeneration across a 40-year time span representative of subjects aged 40 to 80 years old. Our approach provides increased spatial and temporal heterogeneity of the biomarker field and spatial atrophy rate thus providing increased resolution of brain shape changes and a refined approximation of the effect of disease state. We analyze our simulations with respect to *mechanomarkers* that quantify brain shape changes in the form of displacement, area stretch, cortical thinning, sulcal widening, and curvature change. These markers provide insight into the evolving morphology of the brain as it ages and to identify features that are sensitive to early differentiation between healthy and diseased aging. As such, we evaluate our simulations with respect to spatial and temporal differences between disease groups and report years of acceleration in Alzheimer’s disease compared against cognitively normal shape changes as well as cortical and subcortical brain regions that show high sensitivity to early changes between disease groups.

## Methods

In our proposed framework, we solve biomarker progression and corresponding atrophy sequentially in order to increase spatiotemporal resolution of our numerical simulation. In the following, we will introduce (i) the proposed kinetic model for biomarker progression, (ii) its discretization on brain network models, (iii) our constitutive model for cerebral atrophy, (iv) the finite element model used to predict brain shape changes, and (v) our mechanomarkers that quantify shape change-related features. Lastly, we introduce the statistical methods used to evaluate our simulation results.

### Kinetic Model for Aβ and Tau Progression in Neurodegenerative Disease

It has been established that the Fisher–Kolmogorov model is the simplest yet sufficiently accurate model to capture Aβ and tau misfolding associated with neurodegenerative diseases such as Alzheimer’s disease [18]. The Fisher–Kolmogorov model describes the propagation of respective biomarker concentrations and provides the spatiotemporal aggregation of neurotoxic plaques and tangles. It is based on a simple nonlinear reaction-diffusion equation for a single unknown, i.e., in our case the misfolded protein concentration *c* [19, 20]

1 $$\begin{aligned} \frac{\textrm{d} c}{\mathrm {~d} t}=\nabla \cdot ({\boldsymbol{D}} \cdot \nabla c)+\alpha c[1-c], \end{aligned}$$

with the diffusion tensor, ${\boldsymbol{D}}$, that characterizes global protein spreading and the local conversion rate from the healthy to the misfolded state, α. Based on the nature of this equation, once misfolded protein appears anywhere in the brain, i.e., c>0, eventually the whole brain will have misfolded protein, i.e., c=1 everywhere. While this second equilibrium state will likely not be observed in a subject, the simplicity of this model and the low computational cost make it useful to capture the decade-long gradual accumulation of biomarker concentration and serve as a driver or cerebral atrophy. It is important to note here that we chose to model both biomarkers separately without any interactions. While several recent works have started demonstrating how both proteins may be interacting while spreading through the brain [21–26], we opted for this simplifying assumption because of the lack of adequate experimental data to validate protein progression across our observation period of several decades. Future work will have to explore increasingly available longitudinal PET data, e.g., the American Alzheimer’s Disease Neuroimaging Initiative and the Swedish BioFINDER-2 study (NCT03174938), to infer suitable protein interaction models across spatial and temporal scales.

### Discretization of the Fisher–Kolmogorov Model on Brain Network Models

Not unlike prion disease, neurodegenerative diseases are characterized by misfolded proteins spreading from small infected regions to the rest of brain. This process can be modeled as diffusion across a network which can be constructed from the brain’s connectome [27] that is represented as a weighted undirected graph with N nodes and E edges [28]. In the present work, we propose to include 82 regions of interest (ROIs) based on the Desikan–Killiany gyral-based atlas [29] and differentiate between 68 cortical regions (34 in each hemisphere) and 14 subcortical deep gray matter regions (7 in each hemisphere). To ensure that we work with a representative connectome, we use preprocessed data from 100 unrelated healthy subjects aged 22–36 years from the Human Connectome Project [30, 31].

Following work by Fornari et al. [11], we can discretize the Fisher–Kolmogorov model on a network via a nonlinear system of reaction-diffusion equations for the unknown concentrations $c_I$ as follows:

2 $$\begin{aligned} \frac{\textrm{d} c_I}{\mathrm {~d} t}=-\sum _{J=1}^N \,L_{I J} \, {\bar{c}}_J+\alpha \,{\bar{c}}_I\,\left[ 1\,-\,{\bar{c}}_I\right], \end{aligned}$$

where ${\bar{c}}$ is the converged solution of the previous time step, $L_{IJ}$ is the Laplacian that describes the network’s structure, and α is the conversion rate from the healthy to toxic protein configuration. We discretize this model in time using the explicit Backward Euler time integration method and approximate the derivative of *c* with respect to time, *t*, with

3 $$\begin{aligned} \frac{\textrm{d} c}{\textrm{d} t}=\frac{c_{i+1}-c_{i}}{\Delta t}. \end{aligned}$$

We solve the nonlinear system arising at each implicit time step using Newton’s method. Starting from the solution at the previous time step as an initial guess, successive Newton iterates are obtained by solving linear systems involving the Jacobian of the residual, which consists of the discrete diffusion operator and the derivative of the nonlinear reaction term. Iterations are continued until the nonlinear residual falls below a prescribed tolerance. The Laplacian, *L*, in Eq. 2 is defined as $L_{I J}=D_{I J}-A_{I J}$, with adjacency matrix, $A_{IJ}$, and degree matrix, $D_{IJ}$, given by

4 $$\begin{aligned} D_{I J}=\operatorname {diag} \sum _{J=1, I \ne J}^{N} A_{I J}. \end{aligned}$$

Due to distinctly different spreading patterns for Aβ and tau, we distinguish between two adjacency matrices that capture the extracellular propagation of Aβ and the intracellular propagation along the axonal tracts of tau, respectively. The concise forms for the two different adjacency matrices are described in the following two subsections.

#### The Proximity-Weighted Graph

**Fig. 2:**
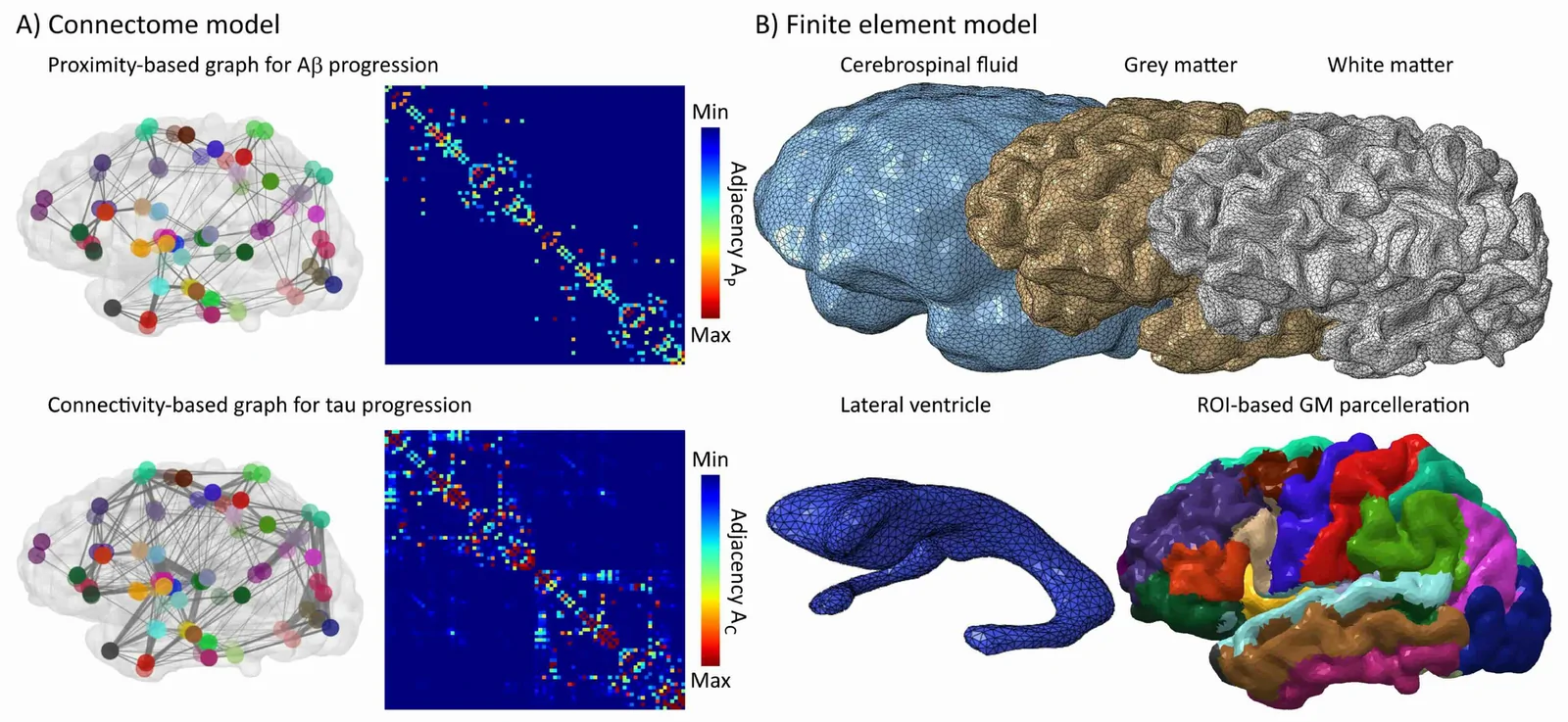
Overview of the computational framework. **A** We use graph models to simulate the prion-like spreading of the neurotoxic proteins Aβ and τ along a proximity- and connectivity-based connectome, respectively. Nodes represent cortical and subcortical regions of interest (ROIs) and edges represent the strength of structural connectivity between ROIs. Corresponding adjacency matrices illustrate connectivity patterns, highlighting stronger intra-hemispheric and sparser inter-hemispheric connections. **B** We use an anatomically accurate finite element model of the brain to produce realistic prediction of brain shape changes associated with cerebral atrophy. Our model distinguishes between cerebrospinal fluid (CSF), gray matter (GM), white matter (WM), and lateral ventricles. GM is further parcellated into 82 cortical and subcortical ROIs based on the Desikan–Killiany atlas

The extracellular spreading of Aβ is associated with the gradual conversion of neighboring healthy protein into the misfolded state and the corresponding growth of amyloid beta plaques. As such, we construct the proximity-weighted graph by identifying neighboring ROIs and then computing the Euclidean distance between their respective centers of mass. The proximity-weighted adjacency matrix, $A_P$, is then assembled as follows [32]:

5 $$\begin{aligned} A_P=\frac{1}{d_{IJ}^2} \text {,} \end{aligned}$$

where $d_{IJ}$ is the Euclidean distance between the centers of mass of regions *I* and *J*. Figure 2A (top) shows the proximity-based graph for Aβ and the corresponding adjacency matrix $A_P$.

#### The Connectivity-Weighted Graph

The intracellular propagation of tau pathology along axonal pathways is appropriately modeled using the brain’s structural connectivity network. Utilizing diffusion magnetic resonance imaging tractography data from 100 subjects in the Human Connectome Project, we generate subject-specific structural connectivity matrices by applying the Spherical-deconvolution Informed Filtering of Tractograms 2 (SIFT2) algorithm [33], implemented in MRtrix3 (Version 3.0.2) [34]. SIFT2 enhances the biological fidelity of tractography-derived connectivity estimates by assigning cross-sectional area weights to individual streamlines, thereby correcting for tractogram reconstruction biases. The resulting connectivity-weighted adjacency matrix, $A_C$, is defined as

6 $$\begin{aligned} A_C = \frac{S_{IJ}}{l_{IJ}^2} \text {,} \end{aligned}$$

where $S_{IJ}$ denotes the SIFT2-weighted streamline count and $l_{IJ}$ the mean streamline length between brain regions *I* and *J*. Figure 2A (bottom) shows the connectivity-based graph for tau with edge line width proportional to edge weight and the corresponding adjacency matrix $A_P$.

### Continuum Model for Cerebral Atrophy

To model the mechanical behavior of the brain, we use the nonlinear equations of continuum theory and introduce the mapping φ from the undeformed, unloaded configuration ${\mathcal {B}}_0$ at time t$_0$ to the deformed, loaded configuration ${\mathcal {B}}_t$ at time t. We adopt the conventional notation, ${\textbf{x}}=\boldsymbol{\varphi }({\textbf{X}},t)$, where ${\textbf{x}}\in {\mathcal {B}}_t$ denotes the position vector in the deformed configuration at time *t* and ${\textbf{X}}\in {\mathcal {B}}_0$ denotes the position vector of the initial configuration at time $t_0$. We characterize local deformations by introducing the deformation gradient, ${\textbf{F}}({\textbf{X}},t)=\nabla _{\!\!X}\boldsymbol{\varphi }({\textbf{X}},t)$, and local volume changes by its determinant, $J=\det ({\textbf{F}})$. Following previous work [13, 35], we model cerebral atrophy as volumetric shrinking and split the deformation gradient into an elastic part ${\textbf{F}}^e$ and an atrophy part ${\textbf{F}}^a$ [17]. The multiplicative decomposition of the deformation gradient, ${\textbf{F}}=\nabla _{\!\!X}\boldsymbol{\varphi }$, yields

7 $$\begin{aligned} {\textbf{F}}={\textbf{F}}^e\cdot {\textbf{F}}^a\quad \text {with}\quad J=J^e\,J^a. \end{aligned}$$

The multiplicative split extends to the Jacobian *J* which breaks down into an elastic volume change $J^e=\det ({\textbf{F}}^e)$ and volume loss by cerebral atrophy $J^a=\det ({\textbf{F}}^a)$. To characterize the hyperelastic material behavior of brain tissue, we adopt the neo-Hookean strain energy density function $\Psi _0$ as the atrophy-weighted elastic stored energy Ψ, which depends exclusively on the elastic part of the deformation gradient,

8 $$\begin{aligned} & \Psi _0=J^a\,\Psi ,\quad \text {with}\quad \Psi =\frac{1}{2}\mu \left[ {\textbf{F}}^e:{\textbf{F}}^e\,-\,3\,-\,2\ln (J^e)\right] \, \nonumber \\ & \quad +\,\frac{1}{2}\lambda \ln ^2(J^e). \end{aligned}$$

Parameters μ and λ are the standard Lamé coefficients which can be expressed via Young’s modulus *E* and the Poisson’s ratio ν in the elastic limit as $\lambda =E \nu /[[1+\nu ][1-2 \nu ]]$ and $\mu =E /[2[1+\nu ]]$. Following arguments of thermodynamics, we can derive the first Piola–Kirchhoff stress tensor ${\textbf{P}}$,

9 $$\begin{aligned} {\textbf{P}}= \frac{\textrm{d} \psi _{0}}{\mathrm {~d} {\boldsymbol{F}}} =J^{\textrm{a}} \frac{\textrm{d} \psi }{\textrm{d} {\boldsymbol{F}}^e} =J^a\,\left[ \,\mu \, {\textbf{F}}^{\textrm{e}}+\left[ \lambda \ln \left( J^{\textrm{e}}\right) -\mu \right] \, {\boldsymbol{F}}^{\textrm{e}\,\textrm{T}}\,\right] . \end{aligned}$$

The Piola–Kirchhoff stress tensor is governed by the quasi-static balance of linear momentum

10 $$\begin{aligned} {\textbf{0}}=\text {Div}({\textbf{P}})+{\textbf{F}}^{\varphi } \text { in } \Omega , \end{aligned}$$

where Ω denotes the domain which is the brain. We assume that we can neglect external body forces ${\textbf{F}}^{\varphi }={\textbf{0}}$. The constitutive form of the atrophy part of the deformation gradient ${\textbf{F}}^a$ is outlined in the following section 2.4.

### Constitutive Description of the Coupling Between Aβ, Tau, and Atrophy Rate

In our multiphysics framework here, we couple biomarker spreading and cerebral atrophy via the atrophy part of the deformation gradient ${\textbf{F}}^a$. More specifically, we pose that ${\textbf{F}}^a$ varies temporally and spatially throughout the brain depending on tissue type and, therefore, is a function of age and both Aβ and tau concentration. We define white and gray matter atrophy to be purely isotropic such that

11 $$\begin{aligned} {\textbf{F}}^a=\root 3 \of {\vartheta }\,{\textbf{I}}\quad \text {and}\quad {\textbf{F}}^e={\textbf{F}}/\root 3 \of {\vartheta }, \end{aligned}$$

where we introduced a measure for volume loss ϑ which is related to cerebral atrophy $J^a$,

12 $$\begin{aligned} \vartheta =J^a\quad \text {and}\quad J^e=J/\vartheta. \end{aligned}$$

Our basic proposition is that cognitively normal (CN) aging is associated with a constant atrophy rate, while both mild cognitive impairment (MCI) and Alzheimer’s disease (AD) are associated with a gradually increasing atrophy rate. More specifically, we rely on previous experimental studies that suggest that both protein biomarkers accumulate following a sigmoidal shape and adopt this here. Based on these considerations, we introduce the two following evolution equations for the atrophy measure ϑ which allows to differentiate between tissue type and state of health,

13 $$\begin{aligned} {\dot{\vartheta }}^{\text {GM}}(t)\,&=\, G_{\text {CN}}\,+\,G_{\text {MCI}}\,\cdot \,{\mathcal {S}}_{A\upbeta }(c_{A\upbeta }(t))\,+\,G_{\text {AD}}\,\cdot \,{\mathcal {S}}_{\tau }(c_{\tau }(t)), \end{aligned}$$

14 $$\begin{aligned} {\dot{\vartheta }}^{\text {WM}}(t)\,&=\, G_{\text {CN}}\,+\,[ \,G_{\text {MCI}}\,+\,G_{\text {AD}}\,]\,\cdot \,{\mathcal {C}}_{\tau }(t), \end{aligned}$$

with rate parameters $G_{\text {CN}}$, $G_{\text {MCI}}$, and $G_{\text {AD}}$ that differ for white and gray matter and brain-level τ load at each time-step, ${\mathcal {C}}_{\tau }(t)$, defined as

15 $$\begin{aligned} {\mathcal {C}}_{\tau }(t) \,=\, \sum _{I=1}^N c_I(t). \end{aligned}$$

Sigmoidal activation functions ${\mathcal {S}}_{A\upbeta }$ and ${\mathcal {S}}_{\tau }$ are given by

16 $$\begin{aligned}&{\mathcal {S}}_{A\upbeta }(c_{A\upbeta }(t))\,&=\,{1}/{2}\,\cdot \,[ \sin {\gamma (c_{A\upbeta }(t)-c_{A\upbeta }^\text {crit})}+1] \nonumber \\&\quad \cdot \delta \langle c_{A\upbeta }(t)-c_{A\upbeta }^\text {crit} \rangle, \end{aligned}$$

17 $$\begin{aligned}&{\mathcal {S}}_{\tau }(c_{\tau }(t))\,&=\,{1}/{2}\,\cdot \,[ \sin {\gamma (c_{\tau }(t)-c_{\tau }^\text {crit})}+1 ] \nonumber \\&\quad \cdot \delta \langle c_{\tau }(t)-c_{\tau }^\text {crit}\rangle. \end{aligned}$$

Here, δ is the switch, i.e., δ=0 for $(c(t)-c^{\text {crit}})<0$ and δ=1 for $(c(t)-c^{\text {crit}}\ge 0$ that activates the sigmoidal transition to the disease-specific maximum atrophy rate and depends on a critical minimal biomarker concentrations $c^{\text {crit}}_{A\upbeta }$ and $c^{\text {crit}}_{\tau }$, respectively. Parameter γ determines the time span over which the atrophy rate transitions to the more severe level.

We use the explicit Backward Euler time integration method to integrate the evolution equation for ${\dot{\vartheta }}$ with

18 $$\begin{aligned} \frac{\textrm{d} \vartheta }{\textrm{d} t}=\frac{\vartheta _{i+1}-\vartheta _{i}}{\Delta t}. \end{aligned}$$

**Fig. 3:**
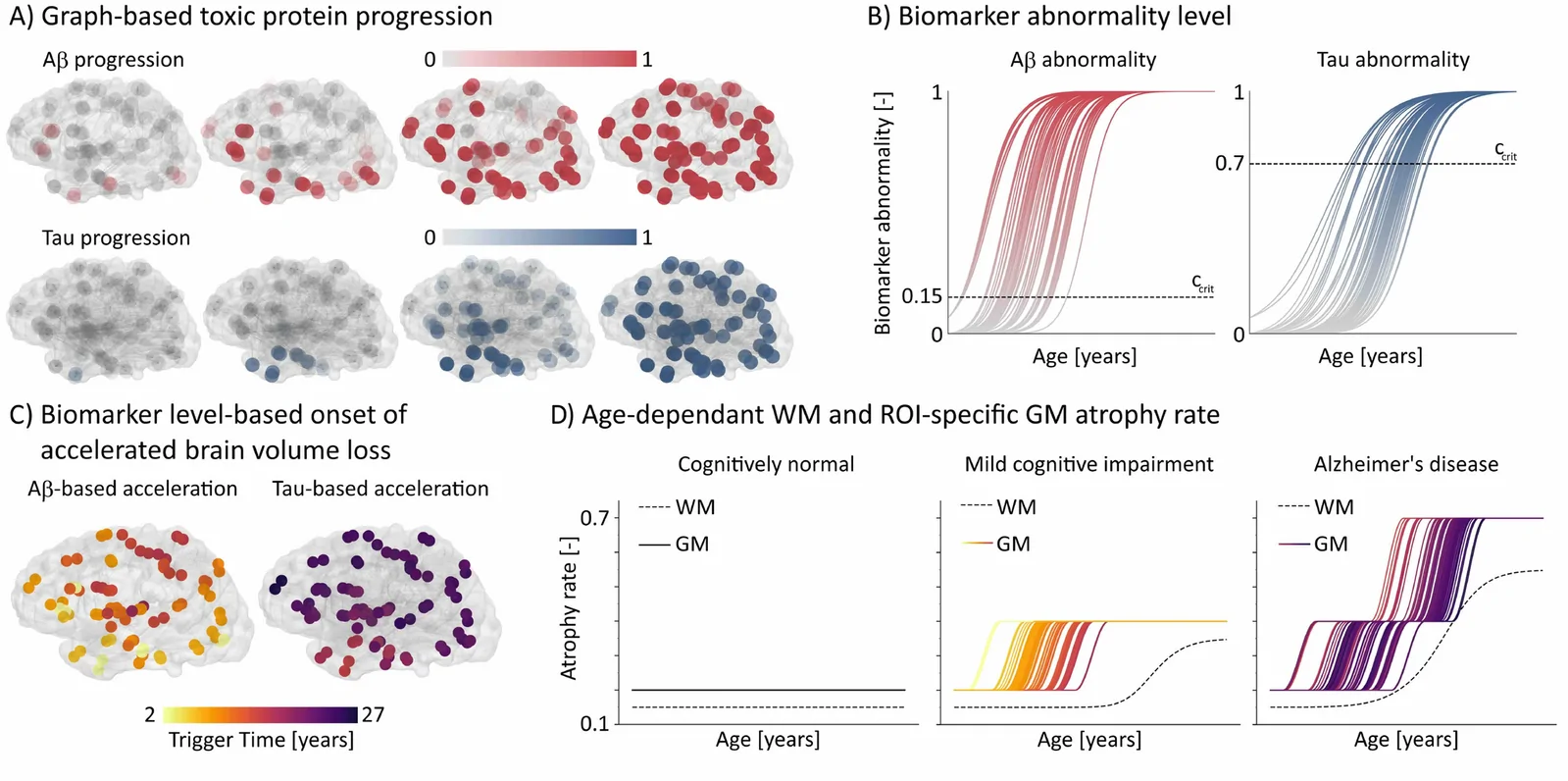
Graph-derived biomarker propagation and its relationship to atrophy rate progression. **A** Spatiotemporal protein progression for amyloid beta (Aβ) based on our proximity model and tau based on our connectivity model. **B** Age-related biomarker abnormality levels of Aβ (red) and tau (blue) for each ROI. Horizontal lines indicate the critical threshold ($c^{A\upbeta }_{crit}$ and $c^{\tau }_{crit}$, respectively) to trigger accelerated atrophy rate associated with mild cognitive impairment and Alzheimer’s disease. **C** Critical biomarker level-based trigger time of accelerated atrophy for both Aβ and τ. **D** Age-dependent WM and ROI-specific GM atrophy rates for the three disease groups, cognitively normal, mild cognitive impairment, and Alzheimer’s disease, respectively

Unlike previous works that only considered a single biomarker (mostly tau) [11, 13, 17], our constitutive model combines the contributions from both biomarker concentrations Aβ and τ based on strong experimental evidence that amyloid beta is a pre-requisite for tau to have its effect on neurodegeneration. As such, the resulting atrophy rates, shown in Figure 3, clearly indicate that Aβ-related atrophy rate changes precede the acceleration of atrophy due to increasing tau concentration. We report specific values for our model parameters in section 2.6.

### Finite Element Brain Model Generation

We use Simpleware ScanIP (Version U-2022.12; Synopsis, Mountain View CA) to segment the T1-weighted magnetic resonance image of a healthy 35-year old adult male adult. Our segmentation differentiates between gray matter, deep gray matter, white matter, the lateral ventricles, and cerebrospinal fluid. To generate a mesh that allows to simulate sulcal widening, i.e., does not have a self-intersecting gray matter surface, we follow the approach presented by Blinkouskaya and Weickenmeier [13]. Specifically, we use grayscale thresholding to isolate the lateral ventricle. We apply isotropic Gaussian filtering (kernel = 3 voxels) to the segmentation to obtain a smooth ventricular surface. Next, we use grayscale thresholding to isolate white matter and apply little isotropic Gaussian filtering (kernel = 1.5 voxels) to preserve the highly folded gray–white matter interface. We obtain the gray matter layer by uniformly projecting the white–gray matter surface by 2 mm. In a last segmentation step, we uniformly inflate the gray matter layer by 5 mm, apply large isotropic Gaussian filtering (kernel = 7 voxels) and then shrink the mask by 3 mm in order to obtain a CSF layer that encapsulates the entire brain and is ultimately used to tether the model in space. We then export the finite element mesh in the Abaqus input file format. When generating the mesh from the tissue segmentation masks, we prescribe a minimum and maximum element edge length of 3 and 3.5 mm, respectively, to ensure a homogeneous regular mesh throughout the brain. The resulting mesh consists of 109,093 nodes and 605,837 linear coupled temperature-displacement elements (C3D4T). The resulting mesh is shown in Fig. 2B. Prior to importing the mesh into Abaqus, we split all gray matter elements into the 68 cortical and 14 deep gray matter regions included in our graph network and create individual element sets in view of transferring data between the graph and finite element model as outlined in Section 2.4. We also subdivide the ventricular surface based on its canonical anatomic divisions to enable spatial analysis. As such, we define three distinct regions: the frontal horn which is the rostral portion of the lateral ventricle extending into the frontal lobe; the posterior body which is the dorsal segment curving over the thalamus; and the temporal horn which is the ventral projection extending into the temporal lobe. This anatomic subdivision aligns with established neuroanatomical boundaries and enables systematic quantification of regional differences in mechanomarkers across disease stages.

### Numerical Implementation and Model Parameters

We solve the biomarker and atrophy problems sequentially. To obtain Aβ and tau concentration for each ROI, we implement an explicit Backward Euler time integration method to solve the network-based biomarker diffusion problem. Specifically, in the case of Aβ progression, we prescribe an initial biomarker concentration of $c_0\,=\,0.06$ across the neocortex, including the lateral occipital, medial orbitofrontal, rostral anterior cingulate, entorhinal, and parahippocampal regions based on well-documented patterns of early Aβ accumulation in early neuropathology [36, 37]; for tau progression, we prescribe an initial biomarker concentration of $c_0\,=\,0.06$ in the entorhinal cortex based on well-established clinical evidence that tau accumulates there first [38, 39]. We define a time step of $\Delta t\,=0.1\,$ and solve a total of 400 iterations to cover an aging period of 40 years. We calibrate our model parameters, i.e., growth rate α and initial concentration $c_0$, by minimizing the error between our graph-derived whole-brain protein concentrations and empirically determined protein load curves derived from PET studies [40]. We simultaneously fitted all three model parameters and obtained a growth rate of $\alpha _{A\upbeta } = 0.38$ and $\alpha _{\tau } = 0.52$ as well as an initial concentration of $c_0 = 0.06$, with a total root mean square error between protein load curves of 0.013.

We use the results from our biomarker progression simulations, to prescribe the atrophy rate curves for each ROI in the continuum model. Specifically, for each ROI, we evaluate Eqs. 13 and 14, to determine the time-dependent atrophy rates for both all gray matter ROIs and white matter. Based on data available in literature [41–43], we selected the disease group-specific atrophy rate constants $G_{\text {CN}}$, $G_{\text {MCI}}$, and $G_{\text {AD}}$ shown in Table 1. For the sigmoidal activation functions, i.e., Eqs. 16 and 17, we select a critical Aβ concentration of $c_{A\upbeta }^{\text {crit}}\,=\,0.15$ and a critical tau concentration of $c_{\tau }^{\text {crit}}\,=\,0.7$, respectively, to reflect the early impact of amyloid beta plaques compared to the delayed tau tangles. The time constant controlling the transition period between stable atrophy rates, γ, is set to 5 years.

**Table 1:** Gray and white matter-specific atrophy rates for our three disease groups, i.e., cognitively normal, mild cognitive impairment, and Alzheimer’s disease

	Gray matter			White matter		
	$G_{\text {CN}}$	$G_{\text {MCI}}$	$G_{\text {AD}}$	$G_{\text {CN}}$	$G_{\text {MCI}}$	$G_{\text {AD}}$
Cognitively normal	0.2	0.2	0.2	0.15	0.15	0.15
Mild cognitively impairment	0.0	0.2	0.2	0.0	0.2	0.2
Alzheimer’s disease	0.0	0.0	0.3	0.0	0.0	0.2

We run all numerical simulations with Abaqus Standard (Version 2024) which uses an implicit solver based on the Newton–Raphson iteration method. In Abaqus, we solve an uncoupled displacement-temperature problem and use the temperature field to prescribe the graph-derived atrophy rates in our atrophy simulations. More specifically, we implement our constitutive model in the user material subroutine, $\textsf{UMAT}$, because it allows not only to compute stress and the tangent but also provides temperature at each integration point. We utilize this feature to encode the graph-derived and ROI-specific atrophy rates for white and gray matter in the temperature field. As such, we prescribe the temperature field via boundary conditions and use amplitude functions to capture the time-dependent atrophy rate increase for the cases of MCI and AD. Moreover, we set conductivity of both tissues equal to 0 to prevent divergence of the thermal problem. Based on our previous experimental work on brain tissue stiffness [44–46], we model white and gray matter tissue to be quasi-incompressible and that white matter is twice as stiff as gray matter. For simplicity, we approximate fluid spaces, i.e., lateral ventricles and the subarachnoid space that is filled with cerebrospinal fluid, as ultra-soft compressive solids also described by a neo-Hookean material model [35]. Additionally, we prescribe zero displacement boundary conditions on the outer surface of the CSF surface to reflect the rigid properties of the skull. These modeling assumptions have proven to be a suitable approximation of not only how the brain is embedded in the skull but also to provide sufficient freedom for the brain to deform during aging and reproduce the characteristic features of cerebral atrophy [13, 35]. Our total simulation time is set to 40 years. All relevant material parameters used in our simulations are summarized in Table 2.

**Table 2:** Summary of our material parameters, including shear modulus μ, Lame’s second parameter λ, density ρ, conductivity *k*, and specific heat $c_p$

	Lamé parameters		Thermal properties		
	μ	λ	ρ	*k*	$c_p$
	[MPa]	[MPa]	[tonne/mm$^3$]	[mJ/(year·mm·K)]	[mJ/(tonne·K)]
Gray matter	0.001	0.032	2E-09	0.0	1E+08
White matter	0.002	0.065	2E-09	0.0	1E+08
Ventricle	0.0001	1.94E-04	2E-09	0.0	1E+08
CSF	0.0005	9.67E-04	2E-09	0.0	1E+08

### Mechanomarkers of Morphological Brain Shape Changes

Cerebral atrophy encapsulates a range of morphological brain shape changes that depend on the severity of neurodegeneration. Quantifying these structural changes provides a series of possible biomarkers to diagnose dementia-type, monitor disease progression, and assess the effect of pharmacological intervention. In the following, we introduce our mechanomarkers.

#### Regional Tissue Volume

To determine regional tissue volume loss, we compute volume by summarizing over all tetrahedral elements that make up respective regions of interest, i.e.,

19 $$\begin{aligned} V_\text {ROI} = \sum _{i=1}^{m} V_i, \end{aligned}$$

where *M* equals to the total number of elements in respective subregions.

#### Area Stretch

For the ventricular and cortical surface, we compute local area stretch, $\lambda _\text {A}$ as the ratio between deformed and undeformed surface area, i.e.,

20 $$\begin{aligned} \lambda _\text {A} = \frac{A_\text {deformed}}{A_\text {initial}}. \end{aligned}$$

Values greater than 1 indicate surface expansion, while values less than 1 indicate contraction.

#### Cortical Thickness Change

We determine cortical thickness by measuring the distance between the pial surface and the white–gray matter interface for a given time-step. We then defined thickness change as the difference between two time points. Following the method used in FreeSurfer [47], thickness is defined as the mean of bidirectional distances:

21 $$\begin{aligned} T = {1}/{2}\,\cdot \,[d_{Pial\rightarrow WM}+d_{WM\rightarrow Pial}], \end{aligned}$$

where $d_{Pial\rightarrow WM}$ and $d_{WM\rightarrow Pial}$ represent the shortest distances from the nodes on the pial surface to the GM-WM boundary and vice versa. For each node, we first identify the closest node on the opposite surface and then compute cortical thickness as the mean of these two distances between the corresponding surface nodes. Cortical thickness change is defined as the difference between the thickness in the undeformed configuration and deformed configuration and is reported at nodal and mean ROI level.

#### Mean Curvature

Pronounced curvature change between two time points is a strong indicator for increased relative deformation of the surface, i.e., increased shear strain in the underlying tissue. As such, we pose that curvature change is a good marker for the mechanical loading of functional surfaces/structures such as the lateral ventricular wall and the gray–white matter interface. To compute a nodal curvature field, we fit a bi-quadratic surface patch to each node and a set number of neighboring nodes (in our case, the nodes from all adjacent elements with connectivity 2) that is parametrized by [48]

22 $$\begin{aligned} z(u,v) = au^2 + 2buv + cv^2. \end{aligned}$$

From this local surface fit, we derive principal curvatures $\kappa _1$ and $\kappa _2$, which are the square roots of the characteristic polynomial $\kappa _{1,2} = a + c \pm \sqrt{(a - c)^2 + 4b^2}$. We then report nodal mean curvature, H, which is given by

23 $$\begin{aligned} H = {1}/{2}\,\cdot \,[\kappa _1 + \kappa _2]. \end{aligned}$$

Ultimately, curvature change, ΔH, is the node-wise difference of the curvature fields between two time points.

#### Sulcal Depth

Sulcal depth is defined as the shortest Euclidean distance from each gray matter node to the convex hull of the brain surface, which serves as an approximation of the outer cortical boundary. As such, sulcal depth $D_i^t$ is given by

24 $$\begin{aligned} D_i^t = \min _{{\textbf{h}}_k^t \in {\mathcal {C}}^t} \left\| {\textbf{x}}_i^t - {\textbf{h}}_k^t \right\|, \end{aligned}$$

where ${\textbf{x}}_i^t$ is the position of the gray matter node and ${\textbf{h}}_k^t$ represents the closest point on the convex hull ${\mathcal {C}}^t$. The computation is restricted to sulcal nodes, identified by negative mean curvature (H<0), as these represent the inward-folding regions of the cortex.

## Results

### How Biomarker Progression Determines Local Atrophy Rate

Figure 3A shows the temporal progression of Aβ and tau concentration across our brain network consisting of 82 ROIs. We observe that the concentration spreads from the respective seeding locations and then follows different pathways based on the two graph structures. Aβ spreads bidirectionally, advancing anteriorly and posteriorly from initial deposition sites toward the precentral and postcentral gyri, with subsequent lateral expansion across cortical regions. In contrast, tau pathology initiates in the entorhinal cortex and spreads along established neural pathways, following intracellular connectivity patterns [49, 50]. Tau first appears in the medial temporal lobe and then progresses in an anteroposterior direction, moving through the temporal lobe and into parietal association areas, eventually reaching the frontal regions. Figure 3B shows the ROI-based sigmoidal biomarker concentration increase with age which clearly follows well-established progression patters from literature [51, 52].

Figure 3C shows the distribution of ROI-wise activation time at which atrophy rate accelerates due to the accumulation of a critical level of Aβ and tau, as described in Section 2.6. We observe an anterior-to-posterior gradient for Aβ and an inferior-to-posterior gradient for tau. The resulting atrophy rate curves are shown in Fig. 3D for the three cases included in this study, i.e., cognitively normal, mild cognitive impairment, and Alzheimer’s disease, respectively. Based on the parameters defined for the present work, for cognitively normal subjects we prescribe two slightly different, yet constant, atrophy rates for white matter and all gray matter subregions; in the case of mild cognitive impairment, we prescribe a white matter atrophy rate that increases with age based on the total biomarker concentration, while the atrophy rate of individual gray matter regions increases according to the curves in Fig. 3D; in Alzheimer’s disease, we obtain atrophy rate curves that show two waves of acceleration based on our proposed coupling of both Aβ and tau. We would like to point out, that according to our model the acceleration in neurodegeneration associated with tau lags behind Aβ-related neurodegeneration by 5 to 23 years depending on ROI, with an average delay of 12.5 years. Ultimately, these three different atrophy rate profiles inherently lead to disease-specific atrophy patterns which we analyze in the next section.

### Morphological Manifestations of Healthy and Accelerated Aging

**Fig. 4:**
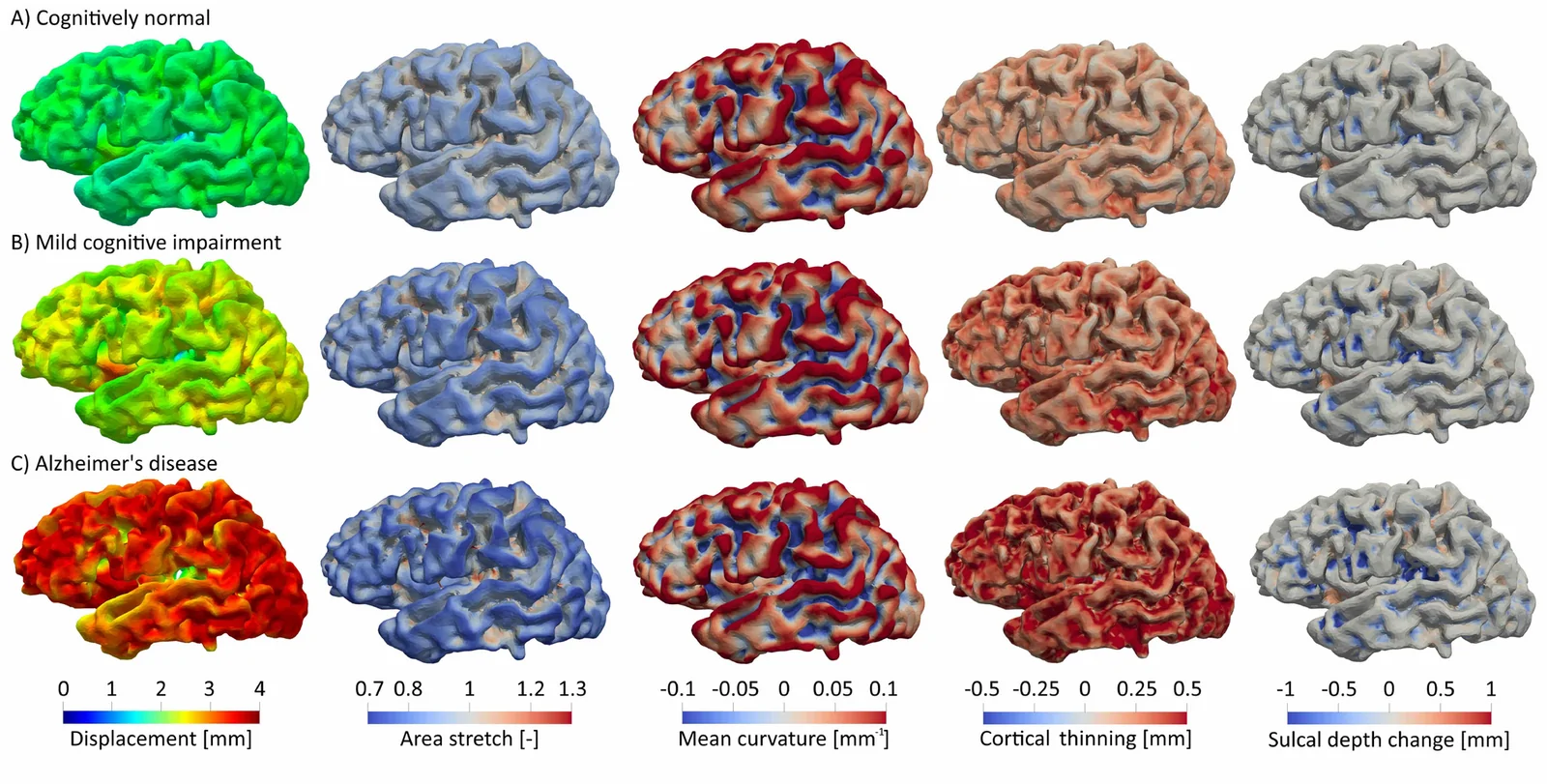
Spatiotemporal patterns of five mechanical biomarkers quantifying brain atrophy on the cortex across the Alzheimer’s disease spectrum: **A** cognitively normal aging (CN), **B** mild cognitive impairment (MCI), and **C** Alzheimer’s disease (AD). From left to right, the mechanomarkers include displacement magnitude (mm), measuring the total tissue displacement from the reference configuration; area stretch (-), quantifying local tissue expansion/contraction; mean curvature ($\text {mm}^{-1}$), capturing changes in surface geometry; cortical thickness (mm), measuring gray matter depth; and sulcal depth (mm), representing the degree of cortical folding. Disease progression correlates with increased severity in all metrics displacement magnitude shows a two-fold increase in AD compared to CN; area stretch reveals progressive tissue contraction; mean curvature demonstrates enhanced folding, particularly at the top of gyri and the pit of sulci; cortical thickness shows accelerated thinning; and sulcal depth decreases consistently. These patterns reveal how pathological neurodegeneration accelerates and spatially modifies normal aging-related morphological changes

Our model predicts that total brain volume decreases by 14.5% in cognitively normal aging, 22.9% in mild cognitive impairment, and 31.2% in Alzheimer’s disease. More specifically, gray matter volume decreases by 15.5% in CN, 26.0% in MCI, and 35.7% in AD; white matter volume decreases by 13.9% in CN, 21.3% in MCI, and 28.8% in AD; and ventricular volume increases by 34.0% in CN, 54.6% in MCI, and 74.4% in AD. The thalamus and the hippocampus are consistently showing maximum volumetric shrinking across disease groups.

Figure 4 shows our mechanomarkers, including displacement magnitude, area stretch, mean curvature, cortical thinning, and sulcal depth of the gray matter surface for the last time point of the three cases A) cognitively normal, B) mild cognitive impairment, and C) Alzheimer’s disease, respectively. Mean cortical displacement magnitude increases from 1.59 ± 0.27 mm in CN to 2.59 ± 0.45 mm in MCI, and 3.64 ± 0.63 mm in AD. That is a 62.51% increase in displacement magnitude from CN to MCI (p < 0.001) and an additional 40.59% increase from MCI to AD (p < 0.001) or 128.47% from CN to AD (p < 0.001).

Average cortical area stretch is 0.97 ± 0.03 in CN, 0.96 ± 0.05 in MCI, and 0.95 ± 0.07 in AD. Moreover, sulcal area stretch is 6.5% higher than gyral area stretch in CN (p < 0.001), 10.3% higher in MCI (p < 0.001) and 14.6% higher in AD (p < 0.001). As such, cortical surface in AD decreases 7.2% more compared to CN and 3.7% more compared to MCI. Figure 6 shows that area stretch is statistically significant between all subject groups (p < 0.001); however, we observe that area stretch is mostly homogeneously distributed across the cortex with no significant regional variation.

Average absolute mean curvature difference is −0.002 ± 0.014 1/mm between CN and MCI, −0.0018 ± 0.011 1/mm between MCI and AD, and −0.003 ± 0.03 1/mm between AD and CN; a statistically significant reduction was only observed between CN and AD (p < 0.05). We also observe an average difference between sulcal and gyral curvature of −0.204 ± 0.00126 1/mm in CN (p < 0.001), −0.207 ± 0.00127 1/mm in MCI (p < 0.001), and −0.209 ± 0.00127 1/mm in AD (p < 0.001). We observe the maximum difference between CN and AD and between MCI and AD in the transverse temporal gyrus. Sulcal values are generally negative numbers across disease groups with −0.1356 ± 0.1762 in CN, −0.1358 ± 0.1732 in MCI, and −0.1355 ± 0.1712 in AD; gyral values are generally positive numbers with 0.0690 ± 0.0532 in CN, 0.0715 ± 0.0580 in MCI, 0.7387 ± 0.0631 in AD. Gyral curvature demonstrates a significant progressive reduction across the disease continuum (p < 0.001 between cognitively normal and mild cognitive impairment; p < 0.001 between mild cognitive impairment and Alzheimer’s disease; p < 0.001 between cognitively normal and Alzheimer’s disease). This decline in gyral curvature represents a mechanical deformation specifically affecting the convex surfaces of cortical folds, while notably, sulcal morphometry shows now statistically significant differences between disease groups, see Fig. 6.

On average, the cortex thins by 0.15 ± 0.09 mm in CN, 0.28 ± 0.14 mm in MCI, and 0.38 ± 0.18 mm in AD. Maximum cortical thinning is consistently observed in the entorhinal cortex, with a difference of 0.19 ± 0.13 mm between CN and MCI (p < 0.001), 0.18 ± 0.15 mm between MCI and AD (p < 0.001), and 0.37 ± 0.28 mm between CN and AD (p < 0.001). Additionally, we observe an average 13.6% difference between gyral and sulcal thinning in CN, of 3.2% in MCI, and of 1.8% in AD (difference is significant in each subject group with p < 0.001 throughout). With respect to sulcal depth, we generally observe a predominant decrease with age on the one hand, and a significant decrease in sulcal depth with disease. Specifically, our model reproduces sulcal widening, i.e., reduction in sulcal depth and increase in sulcal volume, commonly observed in cerebral atrophy. Sulcal depth shows modest but consistent differences between disease groups with an average nodal difference of 3.4 ± 2.1% between CN and AD (p < 0.001) and 1.8 ± 1.1% between MCI and AD (p < 0.001). The largest difference between CN and AD is observed in the cuneus and between MCI and AD in the temporal pole.

**Fig. 5:**
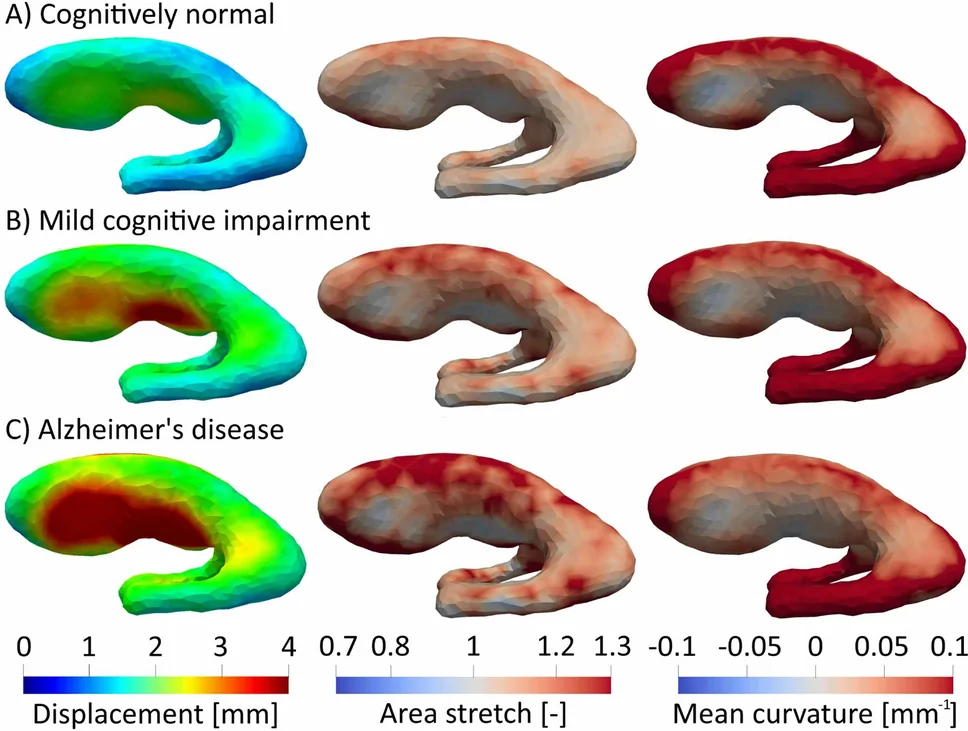
Mechanomarkers of ventricular surface change including displacement magnitude [mm], area stretch [-], and mean curvature [1/mm] at the end of 40 years of aging for **A** CN, **B** MCI, and **C** AD. Displacement magnitude and area stretch progressively increase with disease severity, particularly in the frontal and posterior horns. Mean curvature shows progressive flattening in later disease stages

Figure 5 shows relevant mechanomarkers for the lateral ventricular surface. As such, we report displacement magnitude, volume change, area stretch, and mean curvature. We observe ventricular expansion for all three disease groups with significant differences between them (p < 0.001). Across our 40 year simulation period, ventricular volume increases by 34.0% in cognitively normal aging, by 54.6% in mild cognitive impairment, and by 74.4% in Alzheimer’s disease. Correspondingly, mean ventricular displacement magnitude increases with disease severity. The model predicts an average magnitude of 1.21 ± 0.6 mm in CN, of 1.88 ± 0.95 mm in MCI, and of 2.55 ± 1.26 mm in AD. That corresponds to a 55.53% increase in MCI compared to CN (p < 0.001), 35.63% in AD compared to MCI (p < 0.001), and a 110.94% increase in AD compared to CN (p < 0.001). Regional analysis reveals that the posterior body of the ventricle is the primary site of deformation, with mean displacement increasing from 1.41 ± 0.65 mm in CN to 2.21 ± 1.03 mm in MCI, and 2.99 ± 1.37 mm in AD. These values consistently exceed the frontal horn measurements of 1.32 ± 0.59 mm in CN, 2.04 ± 0.92 mm in MCI, and 2.76 ± 1.23 mm in AD by approximately 6–9% across respective disease stages. Both regions demonstrate substantially greater displacement than the temporal horns (p < 0.001 for all subject groups), which show mean displacements of 0.85 ± 0.28 mm in CN, 1.30 ± 0.43 mm in MCI, and 1.78 ± 0.58 mm in AD.

The ventricle’s average absolute mean curvature difference is 0.02±0.05 1/mm between CN and MCI (p < 0.01), 0.03 ± 0.06 1/mm between MCI and AD (p < 0.05), and 0.005 ± 0.008 1/mm between CN and AD (p < 0.001). Average ventricular area stretch is 1.05 ± 0.04 in CN, 1.06 ± 0.07 in MCI, and 1.07 ± 0.09 in AD. Ventricular surface area increases by 5.8% from CN to MCI, and by an additional 5.2% from MCI to AD. We observe that the anterior horn is the primary site of ventricular expansion, with average area stretch values increasing from 1.09 ± 0.06 in CN to 1.14 ± 0.09 in MCI, and further to 1.17 ± 0.12 in AD, which corresponds to an 4.59%, and 2.63% increase, respectively.

**Fig. 6:**
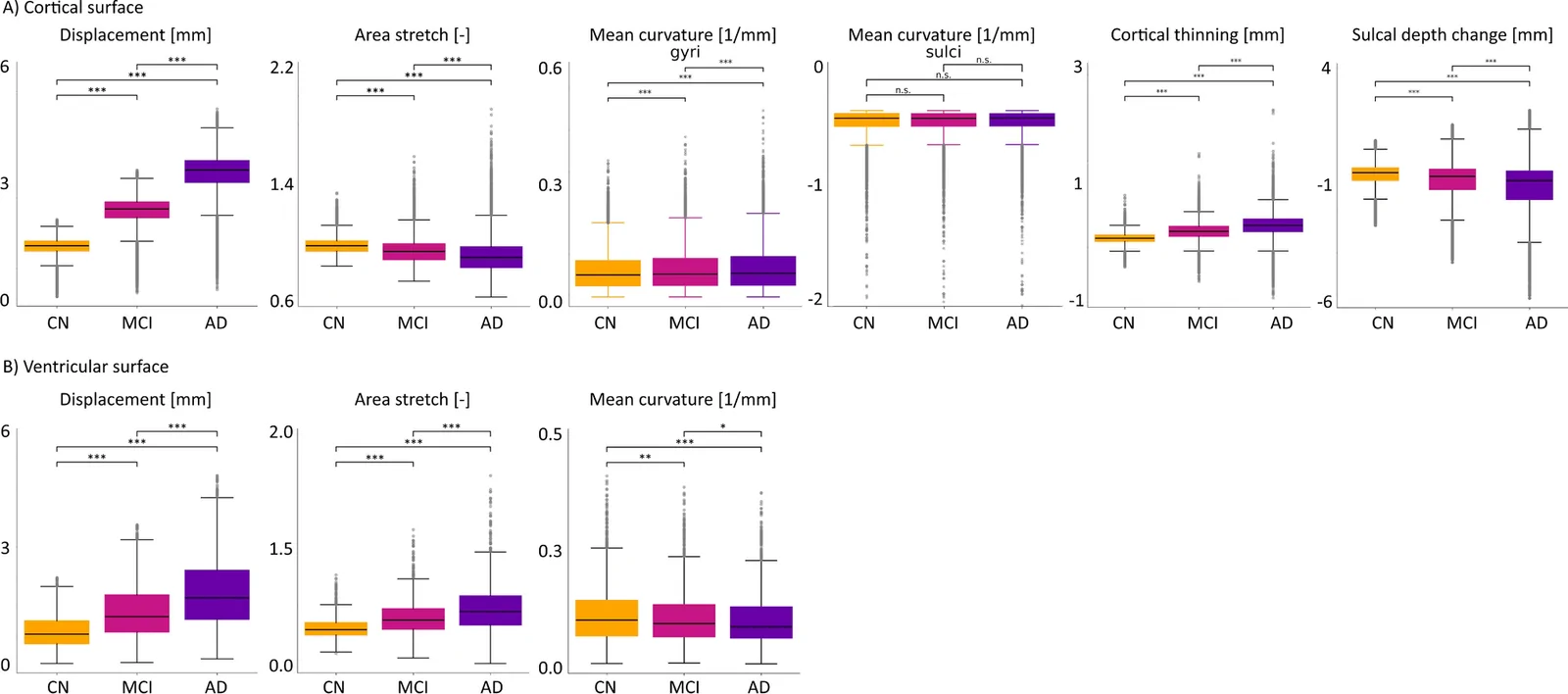
Boxplots of our mechanomarkers for the **A** cortical surface and the **B** ventricular surface. We juxtapose CN with MCI and AD to show how there are consistent trends of the mean value; additionally, we observe the dispersion of each mechanomarker increases with disease state indicating an increasing spatial heterogeneity of shape changes with disease state. We perform a two-sample *t* tests between each two subject groups and report p values of p < 0.05 with *, p < 0.01 with **, and p < 0.001 with ***; n.s. means not significant

To illustrate significant differences between subject groups, Fig. 6 shows the distributions of our mechanomarkers for the a) cortical surface and b) ventricular surface for CN, MCI, and AD. We observe statistical significance between each subject groups for every single mechanomarker (at a minimum p < 0.05 and mostly p < 0.01). For the cortical surface, we generally observe a consistent increase of the mean value between CN, MCI, and AD for displacement magnitude and cortical thinning and a consistent decrease for area stretch and sulcal depth change. For the ventricular surface, mean displacement magnitude and area stretch consistently increase between CN, MCI, and AD, while mean curvature decreases, i.e., the ventricle inflates. Interestingly, however, we observe a progressive increase of each mechanomarker’s dispersion as the disease progresses from CN to MCI to AD. We posit that the gradual increase of the ROI-specific biomarker concentration-dependent atrophy rate, drives spatial variation in mechanomarkers while healthy aging has a rather homogeneous effect on mechanomarkers across the brain.

Overall, we can identify multiple brain regions that consistently experience maximum changes with age and disease. As such, the lateral occipital region shows maximum cortical thinning, sulcal depth change, area stretch, and volume changes in both AD and MCI. The medial temporal, inferior temporal, and entorhinal regions consistently display pronounced area increase and volume change across all disease groups. Interestingly, the frontal pole exhibits maximum values in sulcal depth, area stretch, and volume measurements while maintaining relatively normal cortical thickness. The lateral ventricles consistently show maximum mechanical loading along the edges of the main body as well as the inferior horn while the superior surface of the main body shows negligible changes with age and disease.

### Stress Analysis of the Cortical Surface

**Fig. 7:**
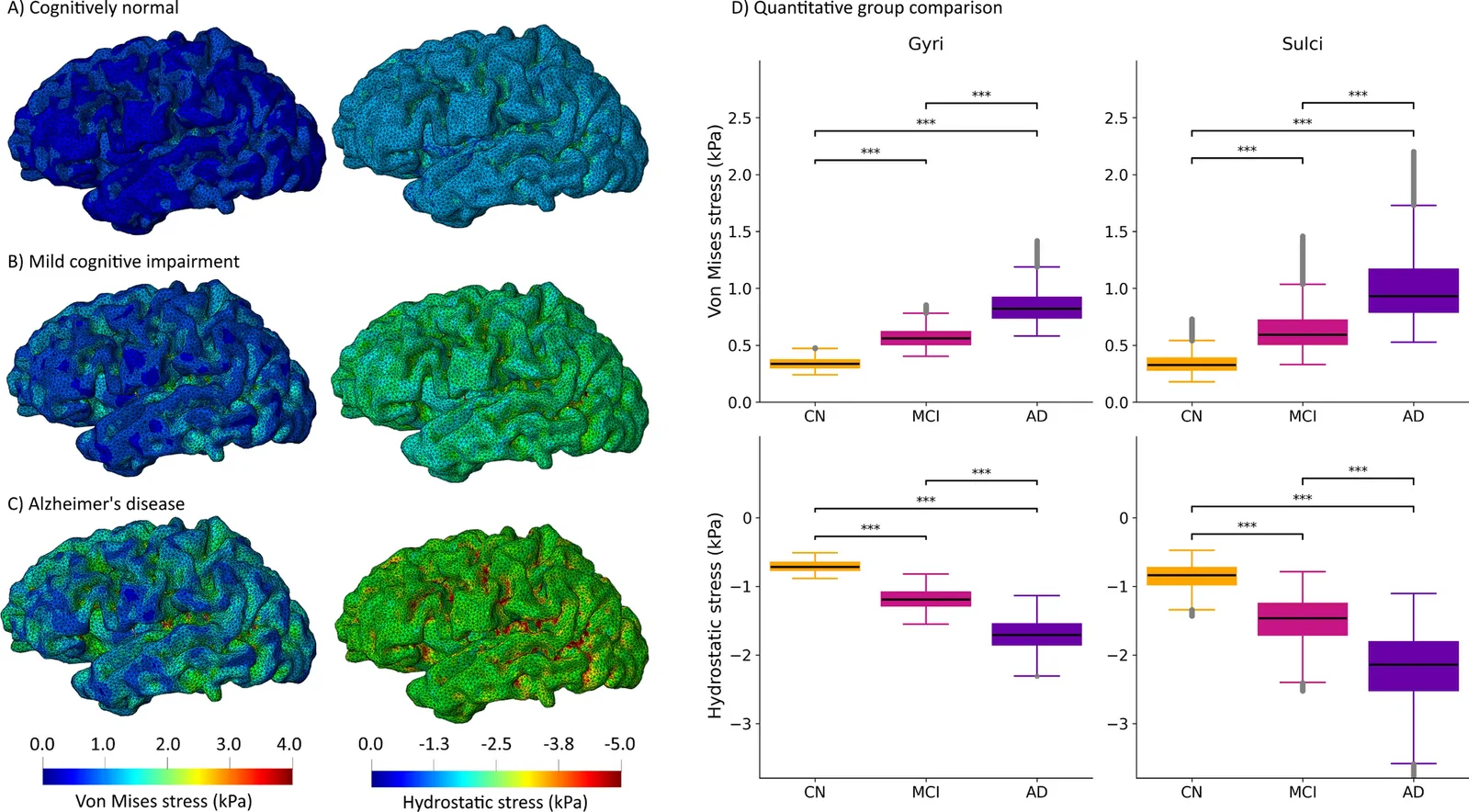
Von Mises stress and hydrostatic stress on the cortical surface in the case of **A** CN, **B** MCI, and **C** AD. Boxplots showing **D** Von Mises stress and **E** hydrostatic stress in sulci and gyri, respectively. We perform a two-sample *t* tests between each two subject groups and report p values of p < 0.05 with *, p < 0.01 with **, and p < 0.001 with ***

Figure 7 shows the von Mises stress and hydrostatic stress on the cortical surface for A) CN, B) MCI, and C) AD. It also shows the distribution of Von Mises stress and hydrostatic stress in gyri vs sulci. We observe that mean von Mises stress increases from 0.35 ± 0.15 in CN to 0.63 ± 0.29 in MCI and 1.00 ± 0.53 in AD; hydrostatic stress increases from −0.86 ± 0.20 in CN to −1.4 ± 0.38 in MCI, and 2.00 ± 0.61 in AD. D) Boxplots reveal statistical significant differences for stress in gyri and sulci (both p < 0.001). Specifically, mean Von Mises stress in sulci is 3.5% higher than in gyri in CN, 14.6% in MCI, and 24.9% in AD, while mean hydrostatic stress is 22% higher in sulci than gyri in CN, 23.4% in MCI, and 29% in AD. These observations agree with recent work from Tueni et al. (2026) that report hydrostatic stresses of the same magnitude [53]. Lastly, we do not observe significant spatial variation between ROIs irrespective of disease state since we do not prescribe region-specific material parameters but simply differentiate between white and gray matter.

### Regional Vulnerability Across Disease Groups

**Fig. 8:**
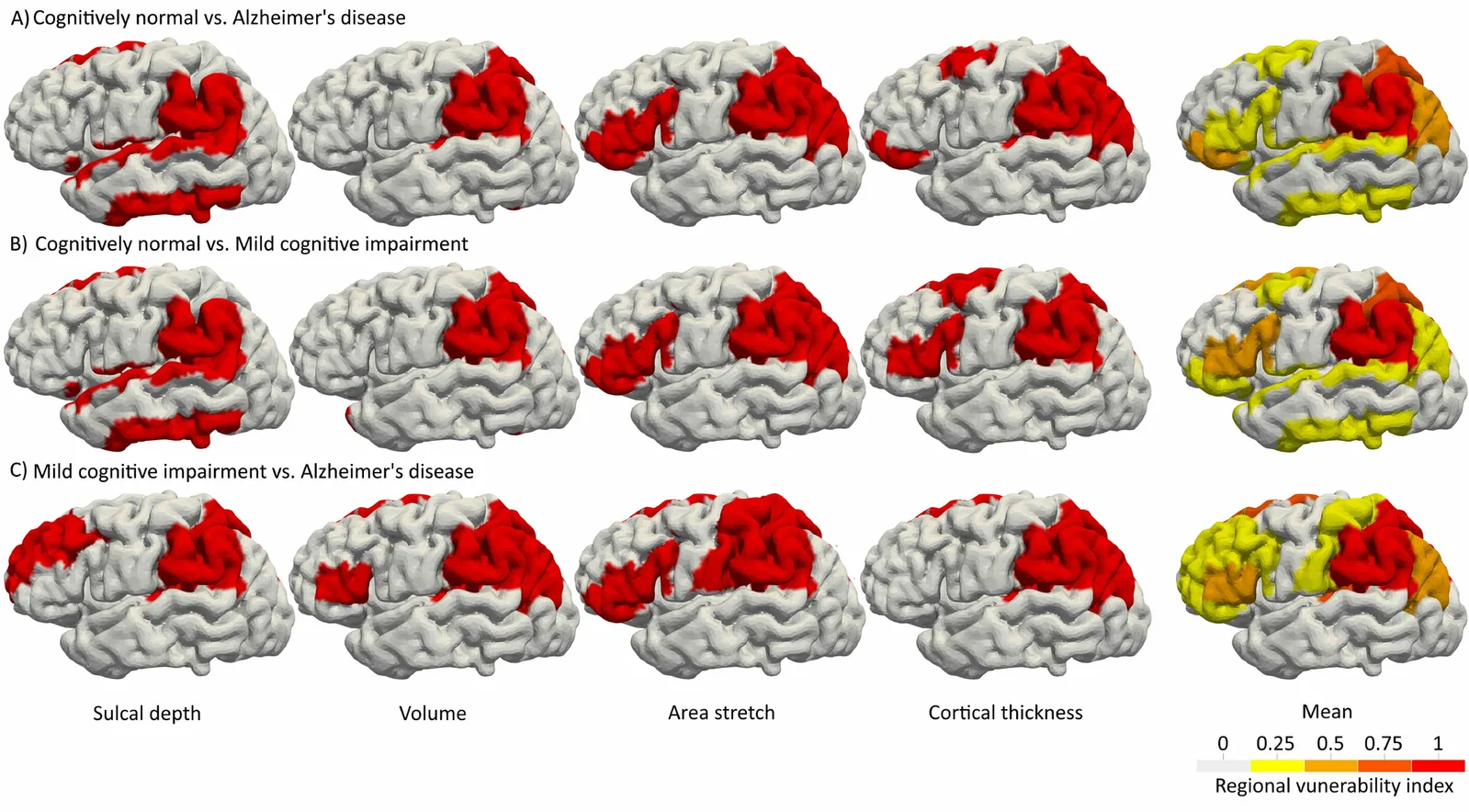
Regional vulnerability analysis between different disease groups. We identified ROIs for which each mechanomarker in the more severe disease group most closely matches the milder final state. Specifically, we compare **A**  cognitively normal and Alzheimer’s disease, **B** cognitively normal and mild cognitive impairment, and **C** mild cognitive impairment and Alzheimer’s disease. The final column shows the averaged field highlighting regions exhibiting high deviations across multiple mechanomarkers

Figure 8 shows regional vulnerability maps that we created to assess regional mechanomarker differences when the more severe disease stage most closely matches the brain of the final presentation of the milder disease stage. More precisely, we first identify the time point at which the more severe disease stage is most similar to the final simulation result of the milder disease stage using the following approach: at each time point and for each mechanomarker field, we compute the node-wise difference between the two models, i.e., we subtract the final output of the milder disease stage from the more severe disease stage. For example, for each time-step, we subtract the final nodal field for CN from the the individual MCI fields; for comparison between CN and AD, we subtract the final output from the CN simulation for the individual AD fields. In the next step, we normalize each mechanomarker’s difference field by the maximum difference value across all nodes and time points. Lastly, we search for the time point at which the total sum of the normalized difference fields across nodes and mechanomarkers is lowest. In the case of CN vs MCI this time point is 28 years, for CN vs AD this is 27 years, and for MCI vs AD this is 33 years. Once these time points are determined, we compute the vulnerability map as follows: for each mechanomarker and ROI, we determine if the mean difference between two disease stages is above the 80th percentile across all ROIs. If so, then we prescribe the value 1 to the ROI; if not, we prescribe the value 0, as shown in the first four columns of Fig. 8. The vulnerability map is ultimately the average of these binarized mechanomarker-wise fields, see last column of Fig. 8, to identify the brain regions that show the most prominent differences, i.e., highest vulnerability, when the overall brain appears to be most similar.

We observe consistent spatial vulnerability patterns for all three comparisons. The comparison between CN and AD reveals that the supramarginal gyrus in the parietal lobe shows the highest vulnerability with differences exceeding the 80th percentile for all markers. The parietal lobe was notably affected, with inferior and superior parietal ROIs showing vulnerability across multiple mechanomarkers. The frontal lobe showed measure-specific vulnerability, with the superior frontal gyrus exhibiting changes in sulcal depth and the caudal middle frontal region showing cortical thickness vulnerabilities. Temporal regions, including superior and middle temporal gyri, demonstrated vulnerability primarily in sulcal depth measurements. The comparison between CN and MCI suggests that similar ROIs in the parietal lobe have elevated vulnerability, the frontal lobe is noticeably more involved. Pars triangularis and opercularis showed pronounced differences in both area stretch and cortical thickness. Temporal regions, specifically superior and inferior temporal areas, showed vulnerability primarily in sulcal depth, similar to the CN-AD pattern. Lastly, the comparison between MCI and AD indicate that most changes occur in the parietal and frontal lobes with little deviation in the temporal lobe. As such, the inferior parietal cortex and supramarginal gyrus exhibit most pronounced differences across all markers, while sensitivity to marker changes decreased for the superior parietal lobule. Overall, the combined vulnerability map in the last column of Fig. 8 confirms the general observation that most marker-based changes focus on parietal and frontal lobules with the supramarginal gyrus showing the highest sensitivity to disease stage changes. The superior parietal lobule shows similar indicative value in undergoing noticeable changes when transitioning from CN to MCI to AD. As such, our analysis suggests that cortical regions in the frontal and parietal lobes undergo pronounced morphological changes as disease severity increases.

### ROI-Based Temporal Offset Between Disease Stages

**Fig. 9:**
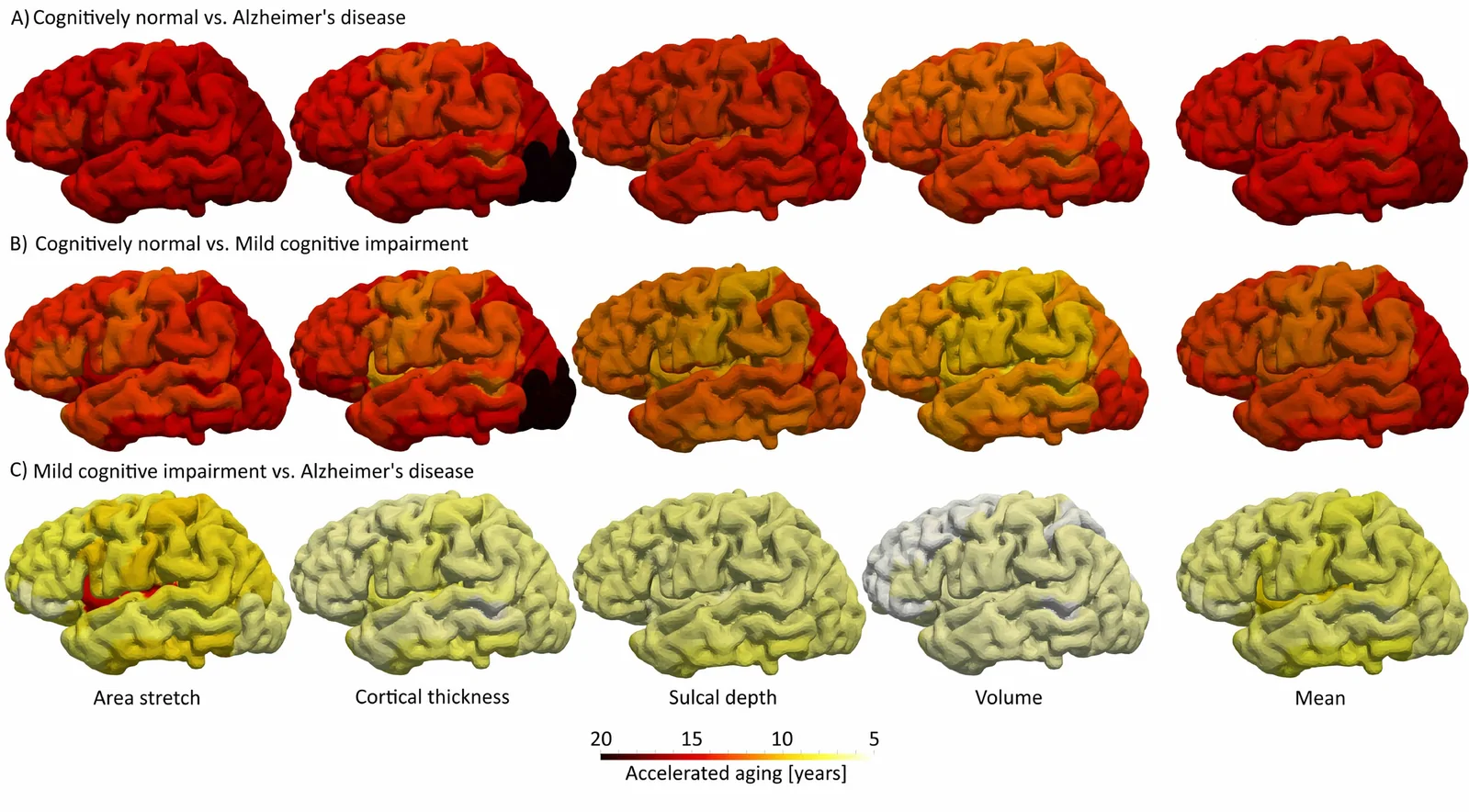
Regional temporal offset analysis across the Alzheimer’s disease spectrum showing accelerated aging patterns. Comparison between **A** cognitively normal and Alzheimer’s disease, **B** cognitively normal and mild cognitive impairment, and **C** mild cognitive impairment and Alzheimer’s disease. Columns 1 through 4 show ROI-based area stretch, cortical thickness, cortical volume, and sulcal depth, cortical volume, respectively. The fifth column is the mean value within each ROI. Darker colors are associated with up to 20 years of acceleration of the more diseased state compared to the less diseased state. Lighter colors are linked to brain regions where mechanomarkers of the two different disease states evolve more similarly

Figure 9 shows the temporal offset between disease stages for each ROI and mechanomarker, i.e., area stretch, cortical thickness, and sulcal depth, volume, respectively. We also compute an averaged offset map for each disease stage comparison, shown in the last column. We construct these maps by identifying the time point at which the more severe disease stages most closely matches the last time step of the milder disease stage and conduct this search for each ROI and mechanomarker. Ultimately, we report the acceleration associated with the more severe stage in years. We clearly see that Alzheimer’s disease is associated with drastically faster brain shape changes than mild cognitive impairment when compared against the cognitively normal brain. We also observe that the acceleration in Alzheimer’s disease compared to mild cognitive impairment is much lower which indicates that it is more challenging to distinguish between the disease stages based on our various mechanomarkers.

The comparison between CN and AD shows an overall average acceleration of 12.06 ± 5.83 years across all regions with the most pronounced accelerations observed in temporal regions, particularly the entorhinal cortex, parahippocampal gyrus, and temporal pole where the well-known onset of amyloid beta accumulation shows an acceleration of up to 15 to 20 years. Across our mechanomarkers, area stretch demonstrates the most significant acceleration in the parahippocampal gyrus by 22 years, the rostral anterior cingulate by 20.2 years, and the entorhinal cortex at 20 years. Cortical thickness reveals the highest acceleration in the entorhinal cortex by 21.4 years, followed by the lateral occipital lobule by 19.9 years, and parahippocampal gyrus by 19.5 years. Sulcal depth exhibits highest acceleration in the entorhinal by 17.7 years, followed by the lateral occipital by 17.4 years, and temporal pole by 16.2 years. Lastly, cortical volume shows more uniform acceleration between 12 and 15 years, with peaks in the entorhinal and parahippocampal cortex of 16 years.

Comparison between CN and MCI generally suggest a similar spatial distribution of acceleration with an overall average acceleration of 10.88 ± 5.31 years across all regions. Area stretch shows the highest acceleration in the parahippocampal gyrus at 21.1 years and the entorhinal cortex at 18.1 years. Cortical thickness follows with the entorhinal cortex at 20.4 years, the lateral occipital at 19.7 years, and the parahippocampal gyrus at 18.7 years. Sulcal depth shows 9–16-year acceleration, strongest in temporal and parietal regions, with occipital involvement, and cortical volumes demonstrate consistent accelerations between 10 and 14 years, with peaks in medial temporal regions at 14 years.

Comparison between MCI and AD shows substantially lower accelerations given the advanced stage of biomarker progression with an overall average acceleration of 6.03 ± 3.02 years across all regions. Area stretch maintains relatively higher values in temporal regions at 14 to 16 years, particularly in rostral anterior cingulate at 15.9 years and entorhinal cortex at 14.2 years. Cortical thickness shows an acceleration of 13 years in the entorhinal cortex, dropping to 6 to 8 years in most other regions, including the lateral occipital. Sulcal depth change shows 10 years of acceleration in entorhinal cortex, with most other regions showing 6–7 years of acceleration. Volume exhibits the most uniform pattern with 5 to 7 years of acceleration.

#### Temporal Sensitivity of Morphological Differences

**Fig. 10:**
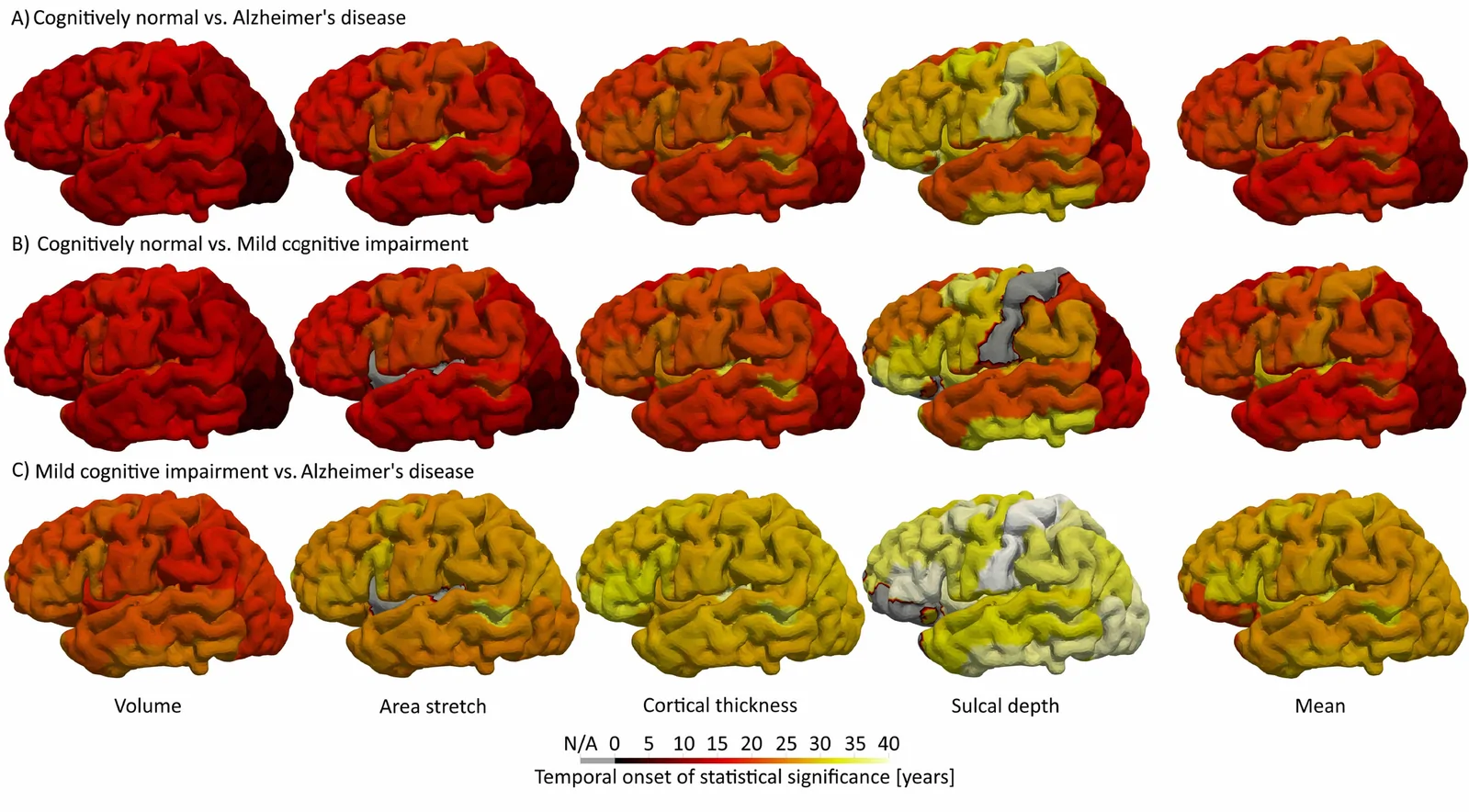
Earliest time point at which statistically significant marker differences (p < 0.05) between models occur for comparisons between **A** cognitively normal and Alzheimer’s disease, **B** cognitively normal and mild cognitive impairment, and **C** mild cognitive impairment and Alzheimer’s disease. Red ROIs indicate early differentiation and yellow ROIs indicate later differentiation. Gray regions indicate that no significant differences are observed within our simulation period. The mean pattern represents the average onset timing across all markers, revealing that structural changes generally initiate in occipital and temporal regions and progress to frontal and parietal areas

Figure 10 shows the earliest time point at which our individual mechanomarkers are statistically significantly different between two disease groups with p < 0.05. More specifically, for each ROI and time step, we perform a *t* test to determine if the mechanomarker of one disease group differs from another disease state. The resulting map provides a spatial indication of brain regions that are sensitive to early shape change differences. The last column shows the averaged onset time across all mechanomarkers and identifies brain regions that could be used to determine disease state as early as possible. Irrespective of individual disease stage, volume and area stretch show the earliest detectable changes, followed by cortical thickness; sulcal depth appears to be less sensitive with some regions never reaching statistical significant differences within our 40-year simulation period.

Our analysis suggests that first significant differences between cognitively normal, mild cognitive impairment, and Alzheimer’s disease emerge as early as 5.5 years in the entorhinal cortex. The rostral anterior cingulate showing first significant changes at 8.75 years and the lateral occipital regions take 11 to 12 years. These are followed by medial orbitofrontal regions at 16 years, then parietal regions at 17 to 20 years, while temporal regions show more heterogeneous patterns at 20 to 25 years. It takes substantially longer for significant differences to emerge between mild cognitive impairment and Alzheimer’s disease, with most regions displaying significant changes after 25 years; that being said, the entorhinal cortex and lateral occipital lobes are most sensitive, with onset times of at 11 to 14 years, respectively.

Volume changes show significant differences the earliest. Our model suggests that the pericalcarine cortex is most sensitive across all disease states, followed by the lateral occipital lobe at 3 to 4 years, and medial orbitofrontal regions at 6 years. The comparison between mild cognitive impairment and Alzheimer’s disease takes on average 7 years longer to show significant differences compared to cognitively normal and mild cognitive impairment.

Area stretch demonstrates early changes in the medial temporal regions, with the entorhinal cortex and lateral occipital lobe showing significant differences at 5 years in both comparisons between cognitively normal and Alzheimer’s disease and between cognitively normal and mild cognitively normal, respectively. Orbitofrontal and parahippocampal regions follow closely just 1 year later. Comparison between mild cognitive impairment and Alzheimer’s disease reveals much later onset times, beginning at 21 years in the entorhinal cortex and progressing to other temporal regions at 22–24 years. Cortical thickness changes emerge first in the lateral occipital lobe at 10 years for both comparisons between cognitively normal and Alzheimer’s disease and between cognitively normal and mild cognitive impairment, respectively. This is followed by superior frontal and medial orbitofrontal regions at 15–16 years. Onset time of significant difference between mild cognitive impairment and Alzheimer’s disease starts much later, with the temporal pole showing the earliest changes at 27 years, followed by widespread involvement of temporal regions at 28 years. Sulcal depth exhibits the latest onset of significant difference among all measures, with initial changes appearing in the lateral occipital lobe, inferior parietal lobe, and medial orbitofrontal regions at 13–15 years when comparing cognitively normal with Alzheimer’s disease. The comparison between cognitively normal and mild cognitive impairment shows a similar but delayed pattern beginning at 14–17 years, while the comparison between mild cognitive impairment and Alzheimer’s disease reveals the latest onset at 27 years in the inferior temporal region.

Overall, ROI level analysis of our numerical simulations reveals statistically significant marker differences between Alzheimer’s disease and cognitively normal brains at on average 18.4 ± 7.5 years, between MCI and normal brains at 16.9 ± 8.2 years, and between MCI and Alzheimer’s disease at 24.9 ± 10.1 years.

### Quantitative Comparison of Our Model Against Imaging-Derived Data

**Fig. 11:**
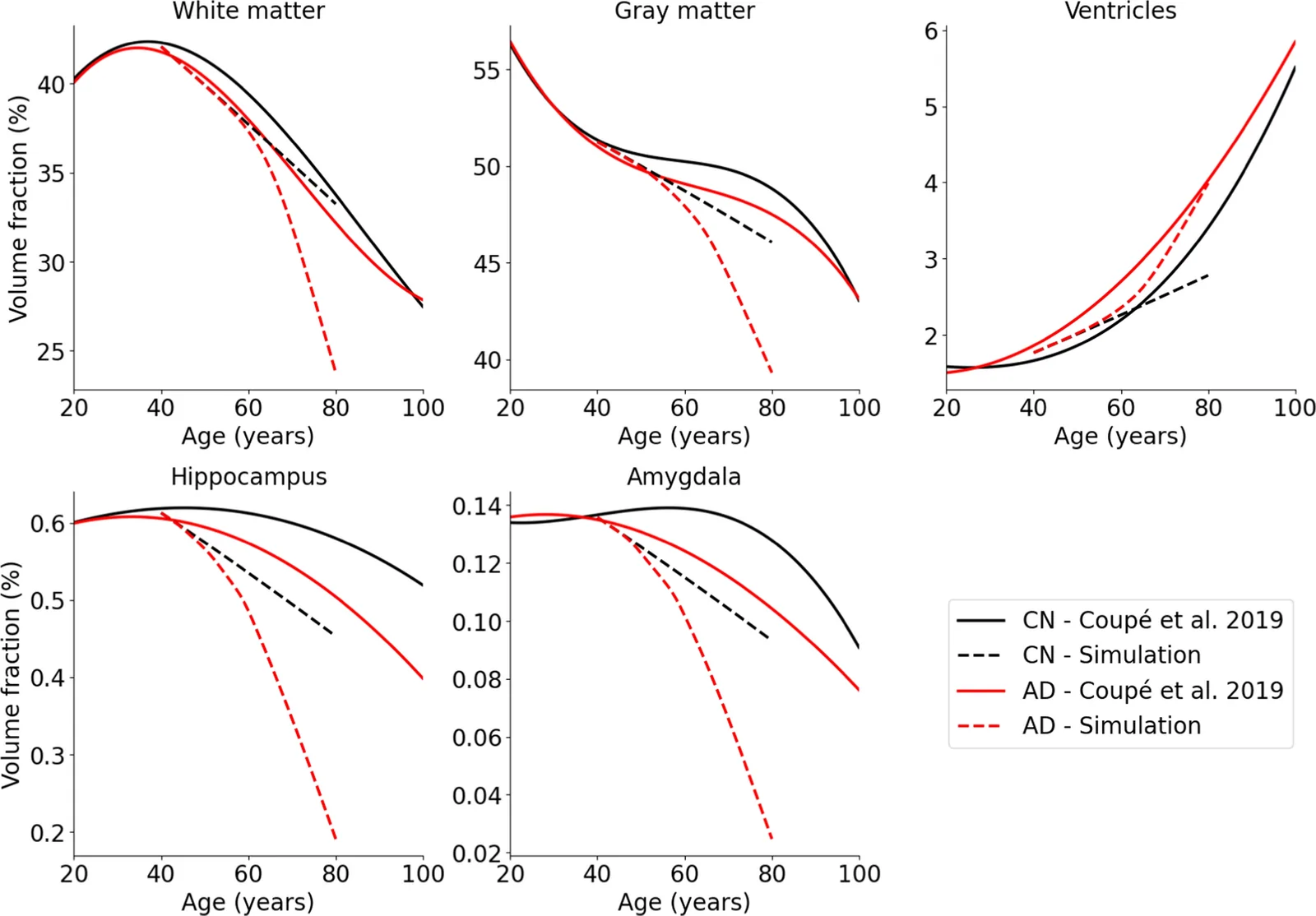
Direct comparison between our aging and AD model against cross-sectional morphometric data from Coupé et al. [54] who quantified brain volume fractions of various cortical and subcortical substructures between the age of 20 and 100 years. Here, we compare volume fractions for healthy and AD white matter, gray matter, lateral ventricles, hippocampus, and amygdala

Validation of our phenomenological disease model and proposed mechanomarkers is generally challenging due to the lack of appropriate data. For example, our finite element model is created from the MNI template atlas for which no longitudinal imaging data exists. Moreover, most longitudinal imaging studies (which would allow to measure longitudinal changes in individual subjects) tend to cover observation periods that are substantially shorter than our simulation time frame of 40 years. Lastly, our proposed mechanomarkers are not commonly reported in literature and only very few studies have quantified shape change features for the brain across multiple disease states. Irrespective of these limitations, we selected two relevant studies for a quantitative comparison with our model. Coupé et al. determined ROI-specific volume fractions in 4329 subjects to determine aging trajectories across the lifespan [54]. While our mechanomarkers focused on the cortical and ventricular surface, we can more easily perform volumetric analysis for individual ROIs. Figure 11 compares data from Coupe et al. (solid lines, black for cognitively normal, red lines for Alzheimer’s disease) with our simulation result. Given inherent difference between brain volumes between their cohort and our template brain, we shifted our curves to the average between healthy and AD volume fraction at age 40 when our simulation starts. We focused our analysis on white matter, gray matter, and ventricles, as well as the hippocampus [55] and amygdala [56] for their highly relevant involvement in early Alzheimer’s disease. Coupé et al. observed early divergence of AD brains from the normal aging trajectory before 40 years of age for the hippocampus and the amygdala [54]. Our model demonstrates a very similar behavior where hippocampus and amygdala split from the healthy aging trajectory nearly from the beginning of the simulation, followed by the ventricle, and only more noticeably for white and gray matter around the age of 60. We generally observe that our model provides an acceptable approximation of healthy atrophy changes (especially for white matter and the lateral ventricles) while we tend to over-predict the atrophy associated with Alzheimer’s disease across all regions except for the lateral ventricles. We would like to point out that we selected atrophy rates that were derived from other morphometric studies that were based on longitudinal imaging data with significant shorter observation periods of about 2–3 years in subjects aged 70–80 years old [41–43]. Since our model considers a constant atrophy rate associated with CN, MCI, and AD, we may overestimate the atrophy during earlier years, i.e., 40–60, thus leading to significantly higher tissue volume loss compared to cross-sectional image data.

**Fig. 12:**
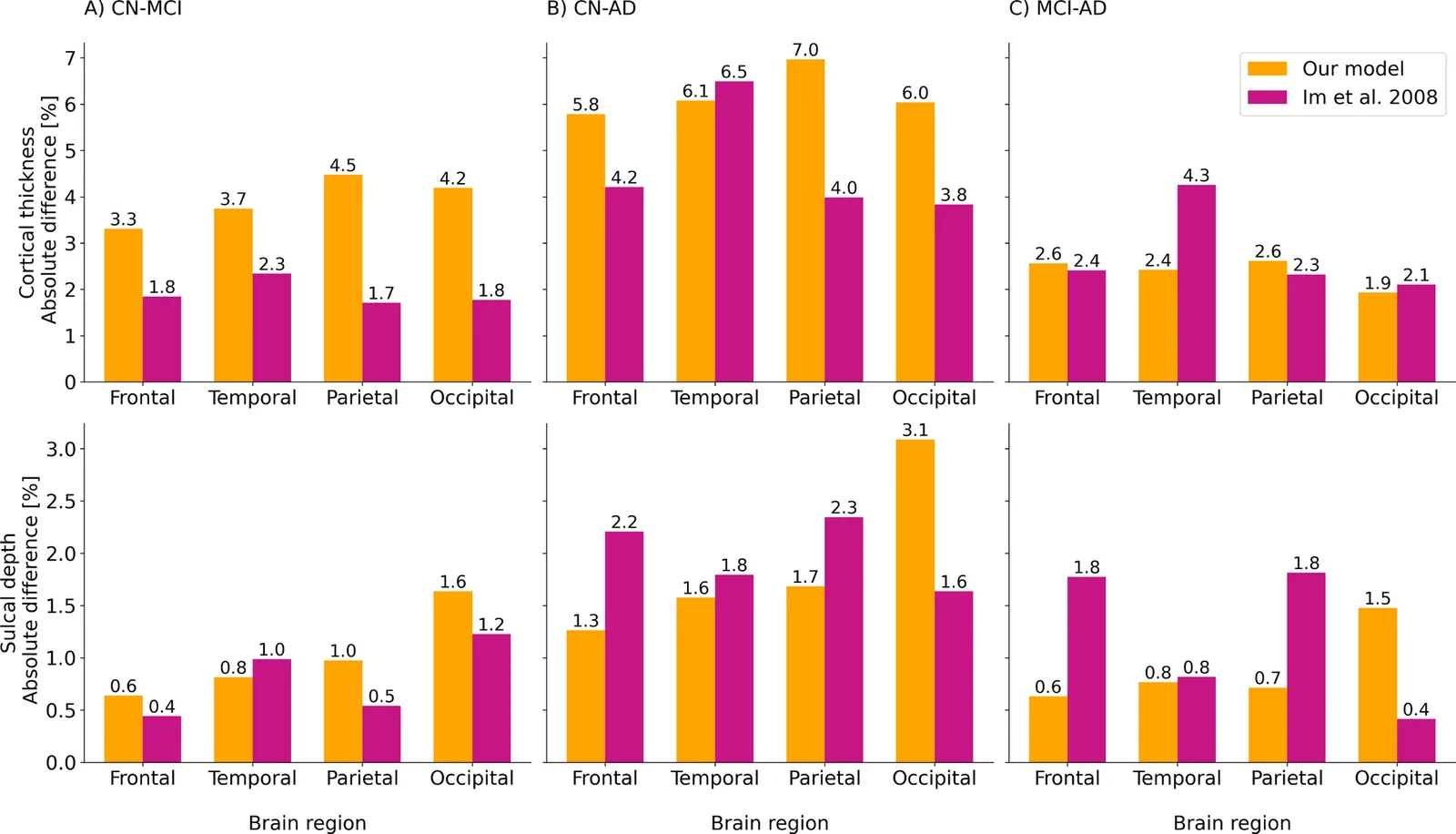
Absolute cortical thickness difference (top row) and absolute sulcal depth difference (bottom row) between **A** CN and MCI, **B** CN and AD, and **C** MCI and AD averaged across the frontal, temporal, parietal, and occipital lobe. We juxtapose data from Im et al. (2008) [57] and our model to gauge the accuracy of our model. We do not expect concise numerical agreement due to major differences between the geometry of our brain model and the data from [57]; however, we consider our results to show close alignment with this experimental data in support of our model

In an effort to validate our mechanomarkers we compare our results against data presented by Im et al. who compared cortical thickness and sulcal depth in CN, MCI, and AD across the frontal, temporal, parietal, and occipital lobes based on 230 subjects aged between 70 and 73 [57]. Figure 12 shows the juxtaposition of our model with data from [57]. To that end, we identified all cortical surface nodes associated with the frontal, temporal, parietal, and occipital lobe, respectively. We then determine mean mechanomarker values for each lobe at age 72 and compute the percent difference between subject groups. Similar to the case of volume fractions, a direct numerical comparison is impossible as we did not run our model on the same subject group, but we observe significant similarities between their data and our model. Most strikingly, our model suggests a larger difference between CN and MCI for cortical thickness compared to Im et al. [57]. Our model and their data are much closer for all other comparisons of both cortical thickness and sulcal depth. We would also like to point out that we observe comparable variation across lobes which we consider to be supportive of our overall modeling approach.

## Discussion

### Neurodegenerative Disease Accelerates Brain Shape Changes by Over a Decade

The temporal dynamics revealed by our multiphysics model offer key insights into the mechanisms by which Alzheimer’s disease pathology accelerates cerebral atrophy compared to cognitively normal aging. Our graph-based biomarker progression model suggests that Aβ-driven neurodegeneration precedes tau-mediated degeneration by an average of 12.5 ± 4.0 years. This finding strongly supports the hypothesis that pathological changes in Aβ level precede those for tau [58]. On the structural side, our shape change-related mechanomarkers indicate that there is an on average 12.06 ± 5.83 year acceleration of significantly different brain shape changes when comparing cognitively normal with Alzheimer’s disease. For mild cognitive impairment, there is a 10.88 ± 5.31 year acceleration compared to cognitively normal and 6.03 ± 3.02 year delay compared to Alzheimer’s disease.

Our findings match well with blood-derived and PET-based studies that observed similar age gaps between measuring critical Aβ and tau levels [59]. For example, Ower et al. observed peak loss of Aβ 8.06 years prior to MCI diagnosis, while tau exhibited peak accumulation 14.17 years following MCI diagnosis [60]. Baek et al. used PET imaging and observed a 16.2-year gap between measuring critical Aβ and tau levels and reported distinct temporal phases where Aβ accumulation stabilizes before the onset of accelerated tau pathology [61], a pattern consistently supported by systematic reviews of fluid biomarkers and neuroimaging studies [58, 62–64].

On the structural level, Gordon et al. reported an approximately decade-long offset in gray matter cortical thinning in Alzheimer’s disease gene carriers when compared to cognitively normal subjects [65]. Coupé et al. provided ROI volume curves for cognitively normal and Alzheimer’s disease subjects across the lifespan [54] and report that healthy and abnormal brain changes start to diverge as early as in the forties. This agrees with our findings that some mechanomarkers show an acceleration by over a decade. As such, area stretch and cortical thickness demonstrate the highest sensitivity, suggesting that surface-based measurements may be more effective in detecting early regional changes compared to volumetric analyses, which show more uniform but less pronounced acceleration. This aligns with studies indicating that surface-based markers capture localized deformation before global volumetric changes become apparent [66, 67]. Notably, our findings align with recent longitudinal studies showing a sequential pattern of structural deterioration, where cortical thickness changes dominate in the early stages of disease progression, while more complex measures like sulcal depth emerge as markers of advanced neurodegeneration [68]. This temporal hierarchy of structural changes suggests that different mechanomarkers may be optimal for tracking disease progression at different stages. Our simulated transition from mild cognitive impairment to Alzheimer’s disease shows a more uniform acceleration of 5–10 years across regions, suggesting a shift from regionally heterogeneous changes in earlier stages to more widespread progression in advanced stages. This pattern is consistent with spatiotemporal analyses of biomarker progression, demonstrating that pathological changes follow a sequential spread throughout the brain [51, 65]. The transition timing aligns with clinical observations, as longitudinal studies estimate an average progression time of approximately 5 years from MCI to AD [69]. A recent longitudinal study has shown that MCI represents a dynamic phase of rapid neurodegeneration characterized by accelerated brain aging compared to cognitively healthy controls. In contrast, AD shows less pronounced rates of further acceleration [70].

### Regions with Early Signs of Accelerated Aging

Ventricular enlargement and associated mechanical loading suggests specific spatial vulnerability patterns, with the highest displacement consistently occurring in regions adjacent to subcortical deep gray matter structures, particularly near the hippocampus. This aligns with longitudinal studies demonstrating that hippocampal atrophy and ventricular expansion, particularly temporal horn enlargement, serve as key markers of Alzheimer’s disease progression and correlate strongly with cognitive decline and disease severity [71, 72]. Additionally, we notice increased mechanical loading along the edges of the ventricle’s main body which spatially correlates with white matter hyperintensity locations. This form of white matter damage is observed in healthy aging and is exacerbated in neurodegenerative disease [73, 74]. From a mechanics perspective the c-localization of white matter hyperintensities and increased mechanical loading of the ventricular surface would suggest that atrophy-driven ventricular deformation is a critical contributor to white matter degeneration in this region of the brain [35, 75].

The distribution of mechanomarkers across the rest of the brain varies between disease groups. More specifically, the comparison between cognitively normal and Alzheimer’s disease reveals peak acceleration in lateral occipital and lateral temporal regions; the comparison between cognitively normal and mild cognitive impairment demonstrates more heterogeneity, with cortical, temporal, and anterior parts of the frontal lobe and posterior parts of the parietal lobe showing the highest acceleration, while the primary motor cortex and somatosensory cortex show the least acceleration as indicated by other studies [70, 76, 77]. The comparison between mild cognitive impairment and Alzheimer’s disease reveals uniform acceleration, with area stretch showing higher acceleration in the insular, parietal, and temporal lobes. Our model’s later-stage involvement of parietal and insular regions is consistent with MRI-based structural progression schemes, identifying these regions as key sites of later degeneration in Alzheimer’s disease [78]. In general, our model predicts a nearly two-fold increase in regional brain displacements in Alzheimer’s disease compared to healthy aging. For example, the insular cortex and areas of the occipital, parietal, and frontal lobes are most affected which aligns with pathological analysis of the spatial distribution of neurodegeneration presented by Bonthius et al. [79]. As such, the insular cortex may serve as a prominent target of AD pathology and exhibit among the highest atrophy rates in Alzheimer’s disease together with the neighboring subregions in the parietal, frontal, and occipital lobes [80]. Our model further suggests that cortical thickness changes most prominently in the occipital and temporal regions for both MCI and, at even increased rates, for AD. This agrees with previous reports on significant parieto-occipital atrophy in Alzheimer’s disease compared to cognitively normal subjects [68, 81, 82].

Area stretch predominantly affects regions known to be vulnerable in early AD, including the medial temporal, inferior temporal, entorhinal, temporal pole, pars orbitalis, and lateral occipital areas [83]. These findings correspond with studies showing reductions in surface area in the precentral gyrus and temporal pole associated with AD risk [84], and significant gray matter reductions in the temporal pole and lateral occipital regions as key markers of disease progression [85]. Sulcal depth measurements on the other hand suggest significant changes in lateral occipital, rostral middle frontal, and inferior temporal regions in line with a surface-based morphometry study on cortical thickness, sulcal depth, and gyrification index within temporal, frontal, and parietal regions [57, 86]. The particular vulnerability of tertiary sulci, as indicated by [87], offers additional support for our findings of region-specific susceptibility to the disease-driven acceleration of aging. The occipital lobe shows strong involvement in transitions from cognitively normal to diseased states but minimal acceleration in Alzheimer’s disease versus mild cognitive impairment suggesting utility as an early marker [81]. In contrast, parietal and insular regions show stronger acceleration in the transition of mild cognitive impairment to Alzheimer’s disease, indicating their role in later disease progression [79]. The distinct regional specificity of many of our mechanical markers that range from early ventricular enlargement and cortical thinning to delayed sulcal changes, suggests the utility of our computational model to detect divergence between healthy aging and pathological neurodegeneration.

### Limitations and Outlook

The most evident limitation of the present work is the need for a more quantitative validation of our constitutive model against available data. As of now, we only perform minimal qualitative comparison against established findings from various longitudinal and population-level imaging studies. Although there is strong support for our computational framework, our proposed coupling between Aβ and tau to drive the spatiotemporal progression of the tissue-specific atrophy rate is only a hypothesis. Additionally, our mechanomarkers are focused on anatomical structures such as the lateral ventricles and the cortical surface and future work should include the analysis of additional cortical and subcortical structures such as deep gray matter and the cerebellum. It is likely that there are other cortical or subcortical regions in the brain that could serve as sensitive markers for accelerated changes due to dementia. And lastly, our entire analysis is based on a single geometry despite considerable inter-subject variability of brain shape, cortical folding, ventricular volume, and many other features. It is important to keep in mind that our predicted brain shape changes are highly dependent on geometry such that the progression of mechanomarkers may vary significantly from another brain shape. Collectively, these limitations warrant additional work focused on uncovering the coupling between Aβ and tau and the mechanomarkers of earliest abnormal brain changes in mild cognitive impairment and Alzheimer’s disease.

We would like to highlight two critical opportunities for future work. For one, we should focus on developing computational methods that allow for inter-subject comparison of our mechanomarkers. Differences in brain geometry between subjects, lack of (node-wise) correspondence between subject-specific finite element brain meshes, and missing methods to accurately morph a template brain mesh onto a subject’s specific brain all hamper cross-sectional analysis. For the other, we should utilize existing longitudinal structural and PET imaging data as a possible avenue to infer both the interactions between Aβ and tau and between the two proteins and cerebral atrophy. By integrating this data in the finite element framework we could then calibrate model parameters, test different constitutive formulations, and ultimately validate simulation results.

Irrespective of these limitations and remaining questions, our constitutive model offers a comprehensive framework to study brain shape changes during healthy brain aging and the acceleration of cerebral atrophy associated with mild cognitive impairment and Alzheimer’s disease. By combining graph-based network modeling and a full-brain continuum model, we offer a solution to introduce spatial and temporal heterogeneity with respect to atrophy rate changes with age. Ultimately, the model’s predicted acceleration of brain shape changes in mild cognitive impairment and Alzheimer’s disease compared to cognitively normal subjects of about 10–15 years is a meaningful result in light of many studies using imaging and blood-derived markers.

## Data Availability

Data will be made available upon reasonable request.
